# The machinery of healthy vasodilatation: an overview

**DOI:** 10.1007/s00424-025-03096-2

**Published:** 2025-06-06

**Authors:** Jana Pourová, Patrícia Dias, Milan Pour, Přemysl Mladěnka

**Affiliations:** 1 Department of Pharmacology and Toxicology, Faculty of Pharmacy, Charles University, Akademika Heyrovskeho 1203, 500 05 Hradec Kralove, Czech Republic; 2 Frick Center for Heart Failure and Arrhythmia, College of Medicine, Dorothy M. Davis Heart and Lung Research Institute, Ohio State University Wexner Medical Center, Columbus, OH USA; 3 Division of Pharmaceutics and Pharmacology, College of Pharmacy, Ohio State University, Columbus, OH USA; 4 Department of Organic and Bioorganic Chemistry, Faculty of Pharmacy, Charles University, Akademika Heyrovskeho 1203, 500 05 Hradec Kralove, Czech Republic

**Keywords:** Vasodilatation, PKA, PKG, NO, EDRF

## Abstract

Cardiovascular function depends on an adequate vascular tone facilitating appropriate blood flow to individual tissues according to their needs. The tone results from the interplay between vasodilatation and vasoconstriction. Its rapid and efficient regulation is secured by many interconnected physiological mechanisms, both at the level of the vascular smooth muscle and the endothelium. The purpose of this review is to provide an update of the current knowledge on the mechanisms of physiological vasodilatation. First, two principal intracellular signaling pathways linked to the activation of protein kinases PKA and PKG are introduced. Subsequently, the role of endothelium-derived relaxing factors together with the endothelium-dependent hyperpolarization is discussed. The roles of ion channels and gap junctions in the communication between endothelium and vascular smooth muscle cells are particularly discussed. Finally, principal vasodilatory stimuli (mechanical, thermal, chemical) and their mechanisms of action are briefly introduced.

## Introduction

The cardiovascular system ensures the transport of blood and the substances dissolved throughout the body. The blood vessels form a vast network, extending to all tissues. This network is not homogeneous; individual vessels differ from each other not only in their diameters but also in their functions and properties. By way of example, the large arteries located close to the heart and distributing the blood pumped are primarily exposed to high pressure and convert the pulsatile blood flow into continuous blood flow. The small vessels play a crucial role in determining the vascular resistance (generally, 60–80% of resistance occurs in the microvasculature), and in regulating the blood flow to individual tissues, thereby significantly contributing to the regulation of the blood pressure. The capillaries secure the exchange of substances between blood and interstitial fluid, and the capacitance vessels control blood return to the heart and function as a reservoir of blood. According to their function, the blood vessels differ, for example, in the elasticity or in the amount of smooth muscle. The basic structure of the vessel wall however remains the same: the endothelium is a single layer of endothelial cells in direct contact with the blood. This is followed by the vascular smooth muscle (VSM) cells and elastic fibers. The outer layer contains fibroblasts, nerve endings, and adipocytes (perivascular adipose tissue, PVAT). The blood capillaries are formed only by the endothelial cells, basal membrane, and the pericytes. The pericytes regulate microvascular tone among other functions (for reviews, see [40, 137, 248]). In brain, the pericytes reflect neuronal activity in the vicinity, and participate in capillary dilatation which can spread back to upstream arterioles (neurovascular coupling). This signaling is complex, and includes the K^+^-mediated hyperpolarization (see Sect. Endothelium-dependent hyperpolarization). The abnormal contractility of pericytes is likely related to the progression of many diseases [232].

The vascular system can respond to immediate needs and regulate the flow of blood through individual tissues. A rapid and adequate vasodilatation or vasoconstriction in individual parts of the vasculature constitute the principal way of blood flow regulation. The VSM plays a crucial role in both processes. Unlike other smooth muscles, this one is significantly influenced by the adjacent endothelium. In general, both the VSM cells and the endothelial cells are influenced by the changes in membrane potential and pH, and by the mechanical, thermal and chemical stimuli. The latter mentioned include both endogenous and exogenous substances.

This review is focused on the mechanism of vasodilatation and attempts to describe its most important components. Other excellent reviews addressing particular aspects of vascular physiology have been published [48, 103, 134, 137, 155, 188, 230]. The aim of this paper is to provide a summary of the machinery of healthy vasodilatation, addressing its principal components and pathways in a comprehensive way. First, an overview of the physiological interplay of ion channels and ATPases in the VSM will be provided. Next, the principal vasodilatory cascades, the Ca^2+^ sparks, and the role of the endothelium will be described. In the last section, the principal mechanisms of action of the vasodilatory stimuli will be briefly addressed.

## Interplay of ion channels and ATPases in the vascular smooth muscle

At the resting membrane potential (roughly − 35–60 mV in vivo, depends on location), there is a low basal movement of ions across the plasma membrane of the VSM cells. The probability of opening of the voltage-gated K_v_, Ca_v_, and Na_v_ channels is low, thus allowing only mild ion flows. The same is true for K_ir_ channels which are also voltage-gated. In general, the closure of the K^+^ channels causes depolarization. The closure can be initiated, e.g., by GPCR agonists on G_q_ receptors. The depolarization opens the voltage-gated Na_v_ and Ca_v_ channels at the plasma membrane, and, in turn, the influx of these ions is initiated. The quick Na^+^ influx increases the activity of the Na^+^/H^+^ exchanger, which further promotes Na^+^ entry, depolarization, and alkalinization. Certain transient receptor potential (TRP) channels are weakly voltage-sensitive [167, 203, 237] and extracellular Ca^2+^ and Na^+^ ions can also enter through them. The Ca^2+^ ions from the intracellular stores flow into the cytoplasm as well, mainly from the sarcoplasmic reticulum (SR). A global increase in cytosolic calcium concentration ([Ca^2+^]_c_) sets the muscle contraction machinery in motion. The calcium-gated channels are opened as a consequence of the increased concentration of Ca^2+^ in their vicinity. The Cl_Ca_ and TRPM_4/5_ channels contribute to further depolarization. In contrast, the K_Ca_ channels (in VSM the big conductance calcium-activated K^+^, BK_Ca_ channels) efflux K^+^ ions which results in the shift of the membrane potential towards the resting values (repolarization). Repolarization closes the voltage-gated channels at the plasma membrane (transition to the long-term inactivated state occurs within milliseconds following membrane depolarization). The Na^+^/K^+^ ATPase secures the efflux of Na^+^ ions from the cell along with the entry of K^+^ ions. If the membrane potential becomes more negative than the resting potential, the K_ir_ channels open and with a mild K^+^ efflux intensify the membrane hyperpolarization until the equilibrium potential of K^+^ is achieved. The [Ca^2+^]_c_ in VSM is reduced to the resting values by the action of the plasma membrane calcium ATPase (PMCA) and Na^+^/Ca^2+^ exchanger (NCX) on the plasma membrane and of the intracellular sarco/endoplasmic reticulum calcium ATPase (SERCA). For review, see [101, 102]. This machinery is supported by the frequent colocalization of the collaborating components; e.g., the vascular BK_Ca_ channels are in the proximity of the Ca^2+^ sources such as the Ca_v_3.2 [88, 89], TRPV_4_ [52] and TRPC_1_ [123] channels. This region is referred to as a Ca^2+^ microdomain and is crucial for the Ca^2+^ signaling in VSM cells (for review, see [215]).

## Vascular smooth muscle in vasodilatation

Vasodilatation involves the relaxation of the smooth muscle in the vascular wall. An increase in the blood flow through the pertinent area is its main consequence. Adequate vasodilatation is an important part of the body homeostasis because it ensures the blood supply and prevents oxygen and nutrient deprivation. In contrast, too extensive vasodilatation may result in a decrease in the systemic vascular resistance and a decrease in the blood pressure. The latter may trigger a reflex response involving, for example, an increase in the heart rate.

Both vasodilatation and vasoconstriction are always active. The resultant vascular tone depends on their extent and the interplay between them at each moment. Imbalance in one or in both can lead to a dysfunction or even contribute to (or cause) a disease. Briefly, the vasoconstriction is terminated by the breakdown of the actin-myosin complex, in which the dephosphorylation of the myosin light chain (MLC) on the MLC_20_ subunit with the participation of the myosin light chain phosphatase (MLCP) plays a key role. Vasodilatation is accompanied by a decrease in the level of calcium in the cytoplasm, due to the efflux of these ions out of the cell or due to the transfer to the SR. As mentioned, the former is mediated by the PMCA and the NCX exchanger, the latter by the intracellular ATPase SERCA. An adequate and fast vasodilatation at the site of need is secured in several ways. At the intracellular level, there are two important vasodilatory cascades associated with the activation of protein kinases A and G. Even though vasodilatation physiologically predominates over vasoconstriction, its magnitude and duration always have to be in line with the acute needs of the particular tissue. Analogously to vasoconstriction, the extent of vasodilatation is regulated by a negative feedback ensuring appropriate scope and transient duration of the response.

The following text will deal with two principal intracellular vasodilating signaling pathways, designated according to their main components: protein kinases A and G. Subsequently, the Ca^2+^ sparks protecting small resistance arteries against excessive pressure will be discussed.

### Protein kinase A

Protein kinase A (PKA, syn. cAMP-dependent protein kinase) is an important cytoplasmic serine/threonine kinase. This protein is a tetramer composed of two regulatory (RI or RII) and two catalytic (C_α_ or C_β_) subunits. The PKA-1 and PKA-2 subtypes are distinguished by the R subunits. The PKA is the major intracellular receptor for cAMP, which regulates many important processes in this way. The A-kinase anchoring proteins (AKAPs) bind the R subunit (preferably RII over RI) and anchor the PKA to the plasma membrane. Simultaneously, they bind other components of this pathway, in particular adenylate cyclase and phosphodiesterase [74]. Also notably, the AKAPs bind other factors associated with vasoconstriction or vasodilatation, including PKC [195].

#### PKA activation

The PKA is activated by cAMP, which is produced by adenylate cyclase (AC). This membrane-bound enzyme is activated mainly by the extracellular vasodilatory substances that bind to their GPCR of the G_s_-type on the plasma membrane. This binding gives rise to the dissociation of the α subunit in the form of G_s_-GTP with the stimulation of AC and formation of cAMP. The G_s_-GTP activity is terminated by the hydrolysis of GTP to GDP through the intrinsic GTPase activity of G_s_; this process is regulated by GTPase-accelerating protein (GAP). The amount of the vascular cAMP is governed by phosphodiesterases (PDE), mainly by the PDE_3_ isoform. The AC can also be activated directly, e.g., by forskolin.

The cAMP causes the dissociation of the catalytic C subunits from PKA. The subunits subsequently phosphorylate the target molecules. This effect is regulated by the amount of the regulatory R subunits. The higher the R:C ratio, the faster the C subunits rebind to the R–C complex [74]. The formation of the R–C complex is promoted by the presence of ATP [139]; consequently, activation of PKA is reduced and vasodilatation counteracted. Simultaneously, ATP inactivates the K_ATP_ channels which promotes the membrane depolarization [117, 189]. The dissociation of the R–C complex is prevented by Ca^2+^ ions, whereas Mg^2+^ ions have the opposite effect. In this context, it is worth mentioning that the PKA anchored by the AKAPs is located close to the Ca_v_ channels [115].

The cAMP acts not only through PKA. The intracellular sensor of cAMP serves as the exchange factor directly activated by cAMP (EPAC). This protein is a Ras-like GTPase that may regulate the activity of Ras homolog family member A (RhoA), the K_ATP_, and the ryanodine receptor operated (RyR) channels (and thus indirectly also the activity of the Ca^2+^-operated channels through Ca^2+^ levels). The effects of EPAC vary in different vessels: in the microvascular VSM cells, it stimulates RhoA and counteracts vasodilatation. The opposite seems to be true in larger arteries [130, 157].

#### Effects of activated PKA

Once activated, the PKA phosphorylates several targets. In general, it is associated with a decrease in the plasma membrane potential (more negative, repolarization), decrease in [Ca^2+^]_c_, dephosphorylation of MLC, and vasodilatation. At the plasma membrane, active PKA (1) increases K^+^ ion efflux through the BK_Ca_, K_v_, and K_ATP_ channels. The latter channels probably open when the resting membrane potential is reached [216]; (2) decreases [Ca^2+^]_c_ by the inhibition of the inositol 1,4,5-trisphosphate receptor operated (IP_3_R) channels and by the activation of SERCA on the SR. The latter is mediated by the phosphorylation of the regulatory protein phospholamban, which is cleaved off [36]; (3) decreases the affinity of MLCK to the Ca^2+^-calmodulin complex (Ca^2+^-CaM) and thus its ability to phosphorylate MLC [38]; and (4) accelerates the hydrolysis of the active RhoA-GTP to inactive RhoA-GDP [163]. In addition, active PKA stabilizes the guanine nucleotide dissociation inhibitor-RhoA (GDI-RhoA) complex by the phosphorylation of GDI and thus prevents the activation of the RhoA/Rho-associated protein kinase (ROCK) cascade [20, 135, 185]. In the smooth muscle of the gastrointestinal tract, the PKA together with PKG reduce the production of diacylglycerol (DAG) and inositol 1,4,5-trisphosphate (IP_3_) by the phosphorylation of PLC_β3_ [165]. Similar effect might occur in the VSM, but no evidence has been reported. A question arises as to whether the PKA directly influences the MLCP. So far, only an indirect effect via the inhibition of RhoA/ROCK has been demonstrated in coronary arteries [256].

#### The PKA feedback

When activated, the PKA exhibits effects that limit its vasodilatory actions described above. It increases [Ca^2+^]_c_ by the activation of (1) the membrane Ca_v_ channels and (2) of the RyR channels on the SR; and (3) weakly activates the MLC_20_ and stimulates Mg^2+^-ATPase activity of myosin independently of [Ca^2+^]_c_. This action is potentiated by the presence of arachidonic acid (at μM levels), which inactivates the MLCP both directly [75] and via ROCK. However, arachidonic acid itself does not influence MLCK [251]. It is the metabolites of arachidonic acid that may participate in its effects. It is notable that, surprisingly, the cAMP (principal activator of PKA) promotes the amplification of Ca^2+^ efflux from the SR (calcium-induced calcium release, CICR) [193].

**Fig. 1:**
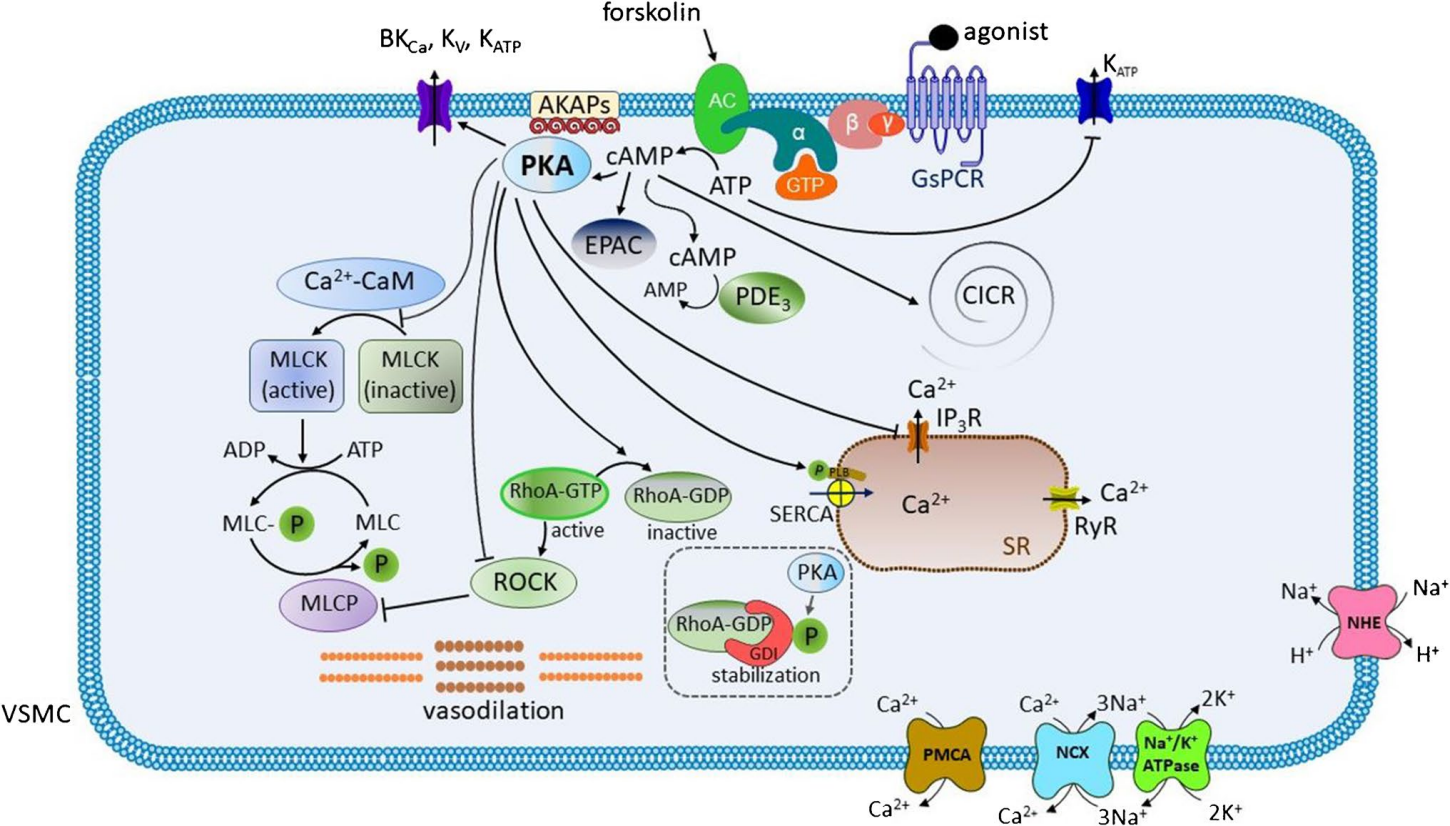
**The role of protein kinase A (PKA) in the vascular smooth muscle (VSM) cell relaxation**. Binding of an agonist to the G protein-coupled receptor of the Gs type (GsPCR) causes the activation of adenylate cyclase (AC), an enzyme that produces cAMP from ATP. The former, in turn, activates PKA. The levels of cAMP are regulated by the action of phosphodiesterases (PDEs) (in the vascular smooth muscle, PDE3 is the main isoform). AC can also be activated directly, e.g., by forskolin. ATP gives rise to the inactivation of the ATP-dependent K+ (KATP) channels, which are important in the maintenance of the resting potential. cAMP promotes the amplification of Ca2+ efflux from the SR (i.e., calcium-induced calcium release, CICR). In addition, cAMP can directly activate the guanine nucleotide exchange factor, EPAC, a Ras-like GTPase that may regulate the activity of RhoA, KATP channels, and, indirectly, the calcium-activated K+ (KCa) channels via the ryanodine receptors (RyRs) and Ca2+ levels. Once activated, PKA (1) increases K+ efflux through the large conductance calcium-activated K+ channels (BKCa), voltage-gated K+ (KV) channels, and KATP channels; (2) decreases the amount of Ca2+ in the cytoplasm by the inhibition of the inositol trisphosphate receptors (IP3Rs) on the sarcoplasmic reticulum (SR); (3) activates sarco/endoplasmic reticulum ATPase (SERCA) through phosphorylation and subsequent cleavage of the regulatory protein phospholamban (PLB); (4) decreases the affinity of the myosin light chain kinase (MLCK) to the Ca2+-CaM complex, thereby decreasing its ability to phosphorylate the myosin light chain (MLC); (5) accelerates the hydrolysis of active RhoA-GTP to inactive RhoA-GDP; and (6) inhibits the Rho-associated protein kinase (ROCK). Thus, PKA decreases plasma membrane potential (becomes more negative), decreases [Ca2+]c, dephosphorylates MLC (through the activation of the myosin light chain phosphatase, MLCP), and promotes vasodilatation. *Other abbreviations: *ADP, adenosine diphosphate; AKAPs, A-kinase anchoring proteins; ATP, adenosine triphosphate; cAMP, cyclic adenosine monophosphate; GDI, guanine nucleotide dissociation inhibitor; GTP, guanosine triphosphate; MLCP, myosin light chain phosphorylated; Na^+^/K^+^-ATPase, sodium/potassium ATPase; NCX, sodium/calcium exchanger; NHE, sodium/proton exchanger; PMCA, plasma membrane calcium ATPase

**Fig. 2:**
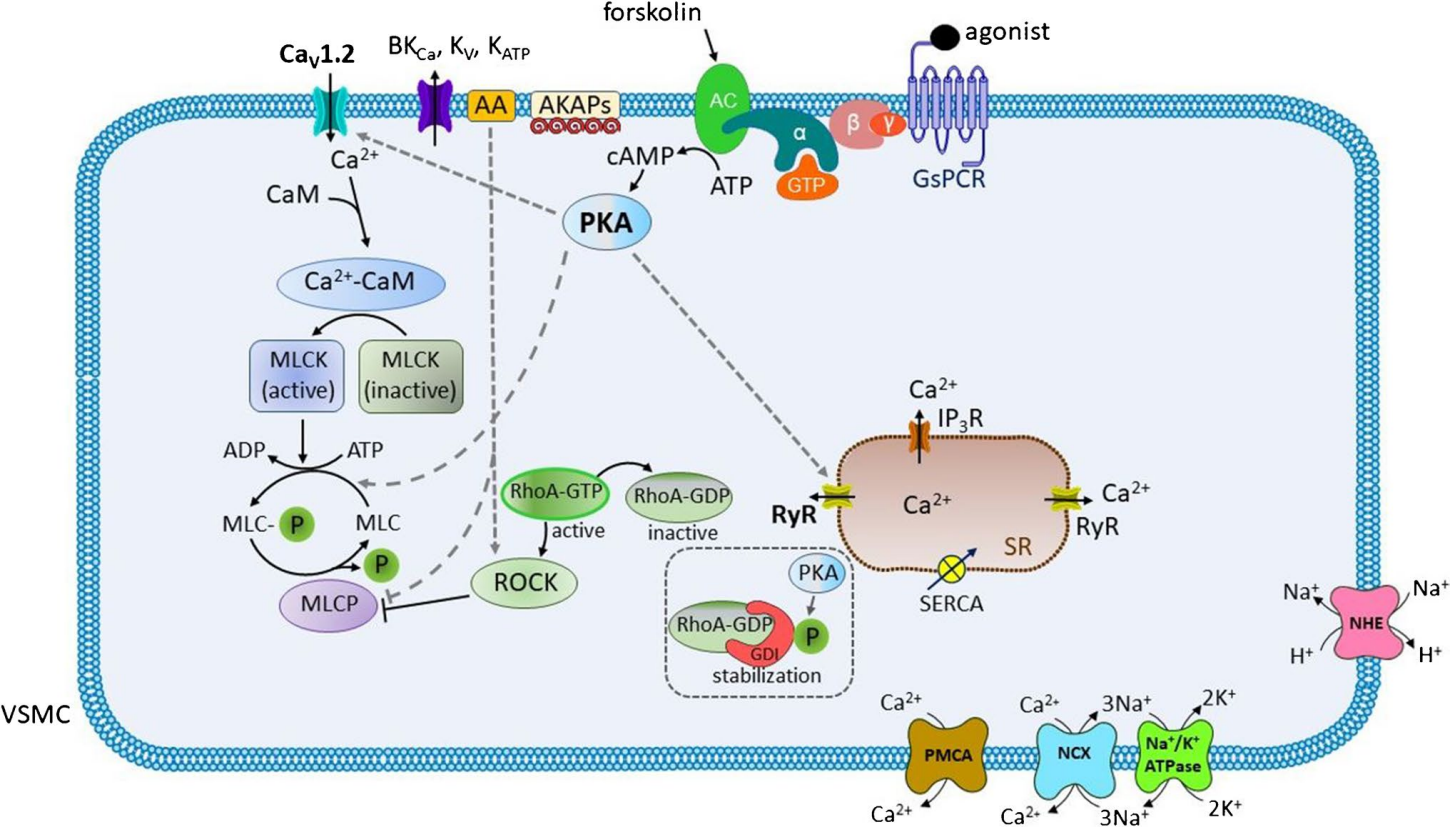
**The dual role of protein kinase A (PKA) in the vascular smooth muscle (VSM) cells. **In limiting its own vasodilatory actions, PKA exhibits the following effects: (1) increases the cytosolic calcium concentration [Ca^2+^]_c_ by the activation of the membrane voltage-gated calcium (Ca_v_) channels; (2) activates the ryanodine receptors (RyRs) on the sarcoplasmic reticulum (SR); and (3) weakly stimulates the myosin light chain (MLC). It is noteworthy that arachidonic acid (AA) may also be involved in the final effect by activating the Rho-associated protein kinase (ROCK) and inhibiting the myosin light chain phosphatase (MCLP). *Other abbreviations*: AKAPs, A-kinase anchoring proteins; ATP, adenosine triphosphate; BKCa, large conductance calcium-activated K+ channels; Ca2+-CaM, complex calcium-calmodulin; cAMP, cyclic adenosine monophosphate; Cav1.2, L-type calcium channels; GDI, guanine nucleotide dissociation inhibitor; GDP, guanosine diphosphate; GsPCR, G protein-coupled receptor of Gs type; GTP, guanosine triphosphate; IP3R, inositol trisphosphate receptor; KATP, ATP-dependent K+ channels; Kv, voltage-gated K+ channels; MLCK, myosin light chain kinase; MLCP, myosin light chain phosphorylated; Na+/K+-ATPase, sodium/potassium ATPase; NHE, sodium/proton exchanger; NCX, sodium/calcium exchanger; PMCA, plasma membrane calcium ATPase; SERCA, sarco/endoplasmic reticulum ATPase

The role of PKA is summarized in Figs. 1 and 2.

### Protein kinase G

Protein kinase G (PKG, syn. cGMP-dependent protein kinase) is a serine/threonine kinase. There are two types, PKG-1 located in the cytoplasm, and PKG-2, which is anchored to the plasma membrane due to myristylation at the N-terminus. In vascular relaxation, the PKG-1 is of higher importance [94], whereas the PKG-2 is important in some tissues, such as the intestine, brain, and kidney [231]. The PKG-1 has two isoforms, PKG-1_α_ and PKG-1_β_.

#### Activation of PKG

PKG is activated mainly by cGMP. The PKG-1_α_ is more sensitive, being activated by about tenfold lower concentration of cGMP than the PKG-1_β_. The cGMP is produced by guanylate cyclase (GC). There two types of GC. The plasma membrane-bound (pGC) is activated by natriuretic peptides [120], while the intracellular form is known as the soluble GC (sGC). It is typically activated by NO from the endothelial cells. Therefore, the sGC is sometimes referred to as an NO sensor [160]. The amount of cGMP is regulated by PDE, principally by the PDE_3_ and PDE_5_ isoforms. The PKG is also activated by cAMP, which acts as a partial agonist. The concentration of cAMP required for PKG activation is about 100 times higher than that of cGMP. Physiological importance is not clear; it might be related to the observation that if cytoplasmic concentration of cAMP increases, the amount of cGMP required for PKG activation becomes higher as well [231, 236].

##### Effects of activated PKG

Once activated, PKG phosphorylates several targets and, similar to PKA effects, the decrease in the plasma membrane potential (becomes more negative), decrease in [Ca^2+^]_c_, dephosphorylation of MLC, and vasodilatation occur. PKG (1) increases efflux of K^+^ ions through the BK_Ca_, the K_v_, and the K_ATP_ channels; (2) inhibits Ca_v_1.2 (L-type calcium channels) and TRPC_6_ channels [217]; (3) activates SERCA on the SR by the phosphorylation of phospholamban [36]; (4) indirectly inhibits the IP_3_R channels by the inositol trisphosphate receptor-associated cGMP-kinase substrate (IRAG) [72, 207]; (5) inhibits the activation of RhoA by its phosphorylation and thereby inhibits the RhoA/ROCK signaling pathway [82]; and (6) phosphorylates GAP and, consequently, stimulates GTPase activity of the G-protein with subsequent reduction of active RhoA-GTP amount [221]. In the rabbit femoral artery, PKG inactivates MLCK and PKC, which decreases the levels of phosphorylated CPI-17 and prevents MLCP inhibition [114]. In the smooth muscles of the gastrointestinal tract, PKG inhibits PLC_β3_ and thus reduces DAG and IP_3_ production [165], and in the gastric smooth muscle cells, the kinase augments MLCP activity through the phosphorylation of the protein M-RIP [143]. A similar mechanism may be functional in the VSM cells.

#### The PKG feedback

To date, little is known about the vasoconstrictive effects of PKG and its negative feedback. Basal NO production (see Section 4.1.) and active NO/sGC/PKG pathway are probably required for RhoA expression and, consequently, to trigger the RhoA/ROCK cascade [200].

**Fig. 3:**
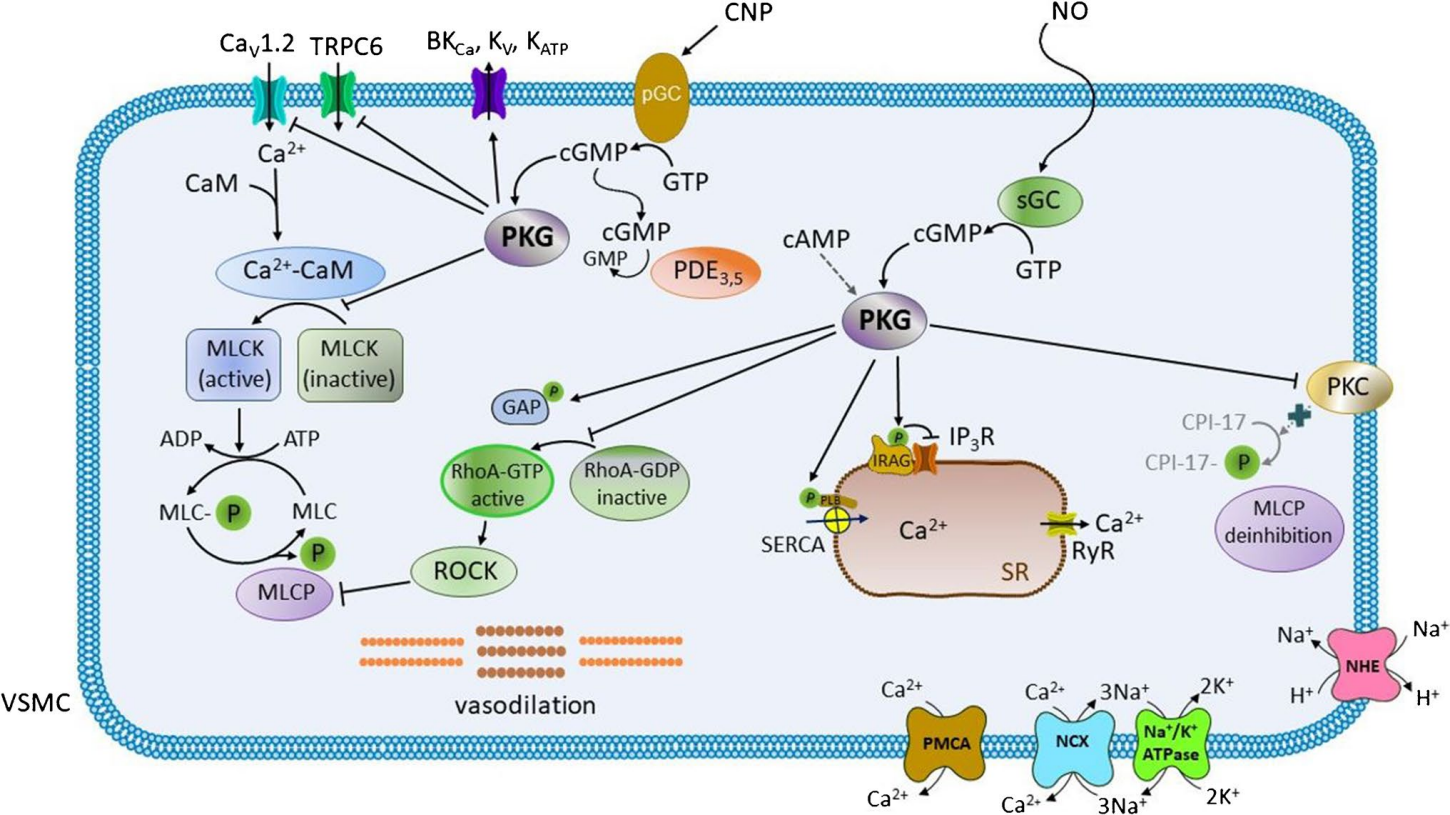
**The role of protein kinase G (PKG) in the vascular smooth muscle (VSM) cell relaxation. **The activation of PKG is mediated by cyclic guanosine monophosphate (cGMP). cGMP is produced by the action of two types of guanylate cyclase (GC): the plasma-membrane bound form (pGC) and the soluble form (sGC). While pGC is activated by natriuretic peptides (e.g., C-natriuretic peptide—CNP), sGC is activated by nitric oxide (NO) generated by the endothelial cells. Upon activation of pGC or sGC, the production of cyclic guanosine monophosphate (cGMP) from guanosine triphosphate (GTP) takes place, with subsequent activation of PKG. The levels of cGMP are regulated by phosphodiesterases (PDEs), with isoforms PDE_3_ and PDE_5_ being predominant in the vasculature. Cyclic adenosine monophosphate (cAMP) can also act as a partial agonist and activate PKG. After activation, PKG (1) further activates the large conductance calcium-activated K^+^ channels (BK_Ca_), the voltage-gated K^+^ (K_V_) channels followed by the opening of ATP-dependent K^+^ (K_ATP_) channels allowing the efflux of K^+^ ions; (2) inhibits the Ca_v_1.2 (L-type) calcium channels and the transient receptor potential cation (TRPC_6_) channels; (3) inactivates myosin light chain kinase (MLCK); (4) phosphorylates the GTPase-activating protein (GAP) with subsequent reduction of active RhoA-GTP; (5) inhibits protein kinase C (PKC) which, in turn, ceases to phosphorylate the phosphopeptide C-kinase potentiated protein phosphatase-1 inhibitor (CPI-17); as a result, the inhibition of the myosin light chain phosphatase (MLCP) is terminated; (6) activates sarco/endoplasmic reticulum calcium ATPase (SERCA) through the phosphorylation of phospholamban (PLB); (7) inhibits inositol trisphosphate receptors (IP_3_Rs) by the inositol trisphosphate receptor-associated cGMP-kinase substrate (IRAG). Thus, PKG activation contributes to VSM cell relaxation. *Other abbreviations:* ADP, adenosine diphosphate; ATP, adenosine triphosphate; Ca^2+^-CaM, complex calcium-calmodulin; GDP, guanosine diphosphate; GTP, guanosine triphosphate; GAP, GTPase-activating protein; MLCP, myosin light chain phosphorylated; Na^+^/K^+^-ATPase, sodium/potassium ATPase; NCX, sodium/calcium exchanger; NHE, sodium/proton exchanger; PMCA, plasma membrane calcium ATPase; ROCK, Rho-associated protein kinase; RyR, ryanodine receptor; SR, sarcoplasmic reticulum

The role of PKG is summarized in Fig. 3.

### Ca^2+^ sparks and waves in the VSM cells

When [Ca^2+^]_c_ in the VSM cells exceeds certain value, the contraction is triggered. Accordingly, the Ca^2+^ ions are usually associated with muscle contraction. However, the changes in the cytosolic Ca^2+^ concentration can be local in microdomains, with negligible alteration in global [Ca^2+^]_c_. The extracellular Ca^2+^ enters principally through the Ca_v_1.2 channels with ~ 75% of the Ca^2+^ influx; the Ca_v_3.2 and the TRP channels play minor roles. This Ca^2+^ entry is the principal source of the initial loading of SR. The Ca^2+^ sparks originate from the SR through the IP_3_R and RyR channels. The sparks produce a local (in the proximity of the plasma membrane) increase in the Ca^2+^ concentration reaching 10–100 µM [56]. This increase activates the adjacent calcium-operated structures, especially the BK_Ca_ channels. Their activation leads to K^+^ efflux which makes the membrane potential more negative and deactivates the neighboring voltage-gated channels [166]. The closure of the Ca_v_ channels closes the cycle of mutual modulation. Co-localization of all participants is necessary. The IP_3_R, RyR, Ca_v_, and the BK_Ca_ channels form functional units [105, 259] that may be located in the membranal *caveolae*. The Ca^2+^ ions are pumped back from the cytoplasm to the SR by SERCA. The local Ca^2+^ levels thus oscillate. The roles of individual channels probably vary according to the type of the artery and species. In large arteries, both the RyR channels and the IP_3_R channels participate, while in the microcirculation, the latter may dominate [240]. Among the RyR channels, the RyR_2_ type may be principal [109]. One Ca^2+^ spark can trigger another; this is referred to as the spark-induced spark activation [191]. The activation can propagate along the VSM cell, and results in a Ca^2+^ wave. This propagation is supported by the CICR, in which the Ca^2+^ ions released from the SR activate further Ca^2+^ release [64]. This process is self-regulating, since from certain Ca^2+^ concentration, the IP_3_R and the RyR channels become closed.

The Ca^2+^ sparks secure important protection of small resistance arteries against excessive pressure. They can be enhanced by various stimuli, including mechanical ones [151], and mere shear stress resulting from physiological blood flow is sufficient. Surprisingly, the AT_1a_ receptors for angiotensin II may be the participating mechanosensors [206]. Accordingly, angiotensin II was reported to stimulate the Ca^2+^ sparks [7]. Importantly, the above mechanism is functional only if the stimulus is appropriately small and the Ca^2+^ increase local. In contrast, the intraluminal pressure above 60 mmHg (observed ex vivo in mice mesenteric artery) leads to vessel contraction, which, in turn, is the consequence of the global rise in the Ca^2+^ cytoplasmic concentration [206]. Finally, in some vessels, the Ca^2+^ sparks result in the activation of the Cl_Ca_ channels, which cause depolarization with subsequent opening of the Ca_v_ channels. This mechanism may play an enhanced role under hypertension [9, 28, 262].

## Endothelium in vasodilatation

The inner lumen of the blood vessels is covered by a single layer of endothelial cells, exposed to a continuous blood flow on one side, and located close to the VSM cells on the other (except for the blood capillaries, which lack the smooth muscle). The endothelial cells are not just a barrier separating the smooth muscle from the blood, and allowing the penetration of chemicals from the blood, since the endothelium can also sensitively detect vasoactive stimuli, and can be, in turn, vasoactive itself. On the luminal surface of many vascular beds, glycocalyx is present. These extracellular polysaccharides form semipermeable barrier, act as shear stress sensors, and influence the NO generation, protect against cell adhesion and infiltration, and against inflammation (for a review, see [63]). The effects of endothelium on the VSM are mediated by chemical mediators and by changes in the membrane potential. While the former are released from the endothelial cells and act on the adjacent cells, the latter act principally via gap junctions (formed by connexins) between cells. This transmission occurs both myoendothelially and between nearby endothelial cells in the axial direction [103]. Potassium cations K^+^ released from the endothelium belong to chemical stimulants, but, in principle, they modify the membrane potential. Similarly, the gap junctions do not mediate just the bidirectional transfer of membrane potential, but possibly also of some mediators (Ca^2+^, IP_3_, small molecules < ~ 1 kDa) [100, 155]. The smaller the vessel, the more important the role of the gap junctions probably is. The vasodilatory substances including the K^+^ ions, produced by the endothelium, are referred to as endothelium-derived relaxing factors (EDRFs). Vasodilatation is also promoted by the endothelium-dependent hyperpolarization (EDH) [69, 113, 235, 243]. In contrast to the VSM, an overall increase in [Ca^2+^]_c_ in the endothelial cells results in vasodilatation precisely due to the production of EDRFs and the induction of hyperpolarization. The opposite effect is promoted by the endothelium-derived contracting factors (EDCFs) and by membrane depolarization. As mentioned above, proper functioning of the blood vessels is conditioned with adequate vasoconstriction and vasodilatation at each moment. If the vascular response is inadequate and endothelium-mediated, endothelial dysfunction is a result. The endothelial dysfunction can be connected to a variety of disorders (for review, see [79, 187]). Impaired production of EDRFs is its main feature. Principal risk factors of endothelial dysfunction are age, obesity, increased oxidative stress, and diseases, such as hypertension and diabetes. In contrast, estrogens prevent endothelium-mediated vasoconstriction [8, 211] and thus promote vasodilatation. Surprisingly, phytoestrogens may relax the cerebral vessels in an endothelium-independent way by the inhibition of Ca^2+^ entry directly on the VSM cells, rather than through the vasorelaxant endothelial mechanisms [226].

Since this review addresses vasodilatation, the principal EDRFs and EDH will be discussed in detail. In contrast to the VSM cells, the formation of EDRFs is started off not only by the activation of the endothelial GPCR of G_s_ type, but, rather surprisingly, also of the G_q_ and G_i_ types. Agonists can originate from the blood, from the surrounding tissues, including autonomic neurotransmitters, or can even be autocrinal from the endothelium. Some physical stimuli display similar effects. The activation of the GPCR activates cellular pathways, and the activated kinases subsequently phosphorylate their target proteins. As a result, the production of EDRFs and the modification of the membrane channel activity follow. Some vasoactive stimuli may activate the endothelial membrane channels directly. Once released from the endothelium, EDRFs both influence the neighboring cells and act in an autocrine manner. Some of their effects are manifested on the cell membrane, others are intracellular. K^+^ ions play an important role. Their efflux decreases the membrane potential (becomes more negative) which is propagated by myoendothelial (MEGJ) and endothelial (EEGJ) gap junctions. The neighboring cells are simultaneously influenced by the increase of K^+^ concentration in the adjacent extracellular space. NO has long been considered as the most important EDRF and has thus been the subject of intense research. However, recently published data show that NO may be the dominant EDRF only in large arteries, while in smaller resistance arteries, most likely EDH, is probably principal. The next sections summarize the data on the most important EDRFs: NO, H_2_O_2_, H_2_S, CO, derivatives of arachidonic acid, and some others.

### NO

In the blood vessels, nitric oxide is formed from *L*-arginine by the constitutive endothelial NO synthase (eNOS) together with *L*-citrulline. The eNOS additionally produces the superoxide anion O_2_^•− ^[229].

#### eNOS

Inactive eNOS is located in the plasma membrane invaginations, *caveolae*, anchored by myristoylation, which secures colocalization with other structures. It is also in the Golgi complex, but its capacity to generate NO is blunted there. The *caveolae* formation is associated with specific proteins (the small integral membrane proteins caveolins, filamentous peripheral membrane proteins cavins, Eps15 homology domain) and with membrane lipids including cholesterol [111]. The inactive eNOS is bound to caveolin-1 and becomes activated following caveolin-1 displacement by the Ca^2+^-CaM complex. Various cofactors are required for the NO production, such as tetrahydrobiopterin (BH_4_), flavin adenin dinucleotide, flavin mononucleotide, and heme [5, 229]. Optimal BH_4_ concentration is of special importance, since BH_4_ mediates the dimerization of eNOS, necessary for NO production. Under the eNOS uncoupling, the resultant monomers preferentially produce superoxide anion O_2_^•−^, which can be converted to another important EDRF, H_2_O_2_ (see Sect. H_2_O_2_) [46, 186]. The non-caveolar caveolin-1 domains or scaffolds also exist indicating a possible non-caveolar association with eNOS. However, these domains are structurally and functionally distinct from *caveolae* as reported for the caveolin-1 signaling [106, 131, 213, 261].

The activity of eNOS is effectively regulated by posttranslational modifications [34, 43, 228], and by the heat shock protein 90 (HSP90). Generally, they modify the sensitivity of eNOS to the Ca^2+^-CaM complex. The eNOS can be posttranslationally phosphorylated on tyrosine, serin, and threonine. The NO production is augmented when Ser-1177, Tyr-81, Ser-615, and Ser-633 are phosphorylated, and Thr-495, Tyr-657, and Ser-114 dephosphorylated. The phosphorylation of Ser-1177 and Thr-495 can even switch between these two positions [154, 235], and Thr-495 phosphorylation may regulate the ratio of NO and superoxide produced [91]. Many kinases participate in the phosphorylation of eNOS, among them the calcium-calmodulin-dependent protein kinase II (CaMKII) [61], PKA, protein kinase B (PKB, Akt), PKC, AMP-activated protein kinase (AMPK), and ROCK [43, 91, 154]. However, the possible link between eNOS phosphorylation/dephosphorylation and its activity has not been fully clarified, as reported recently for Ser-1177 [54].

The eNOS can also be posttranslationally S-nitrosylated, acetylated, acylated, glycosylated, and glutathionylated [91]. The S-nitrosylation of Cys-94 and Cys-99 decreases the enzyme activity and can be regarded as an autoregulation of NO production. Acetylation both decreases (Lys-494 and Lys-504) and increases (Lys-609) the activity of eNOS. Interestingly, the Lys-609 acetylation can be induced by acetylsalicylic acid and this effect is independent of its cyclooxygenase inhibition [107]. The acylation of eNOS also mediates its localization in the *caveolae* [91] (notably, from a chemical point of view, acetylation is a form of acylation). Glutathionylation of Cys-689 and Cys-908 leads to uncoupling of the dimer and superoxide production, and can be reversed by the AT_1_ blocker, telmisartan [116]. The HSP90 facilitates caveolin-1 displacement by the Ca^2+^-CaM complex [77] and enhances the interaction of eNOS with PKB [62]. This process modulates the folding of eNOS, regulates hem insertion into the immature enzyme [16], and protects against calpain-mediated degradation [11]. NO production is further influenced by other factors, such as the eNOS traffic inducer (NOSTRIN) and the eNOS interacting protein (NOSIP), which support the translocation of eNOS from the *caveolae* to the intracellular region. Interestingly, NOSTRIN was reported to interact directly with the membranous cholinergic M_3_ receptors [119].

#### Production of NO

The production of NO can be stimulated in two ways, typically by an increase in [Ca^2+^]_c_ and direct activation of eNOS by the Ca^2+^-CaM complex. This cascade can be triggered by the agonists on the GPCR of the G_q_ type, which initiate the transfer of both sarcoplasmatic and extracellular Ca^2+^ into the cytoplasm. Alternatively, the [Ca^2+^]_c_ remains at the resting level, and NO production increases with the rise in eNOS sensitivity to the Ca^2+^-CaM as a result of posttranslational modifications with the participation of HSP90. The fluid shear stress can serve as an example. When detected by the endothelial mechanosensors, the phosphatidylinositol-4,5-bisphosphate 3-kinase (PI_3_K) is activated. Consequently, kinases PKB [60] and PKA [19] are activated and these enzymes further phosphorylate eNOS on Ser-1177.

The production of NO is regulated by kinases and phosphatases in both positive and negative ways. The [Ca^2+^]_c_ increase activates the dependent kinases, including CaMKII and PI_3_K, which, in turn, activate other kinases, such as PKB. Accordingly, the vasodilatory effect of bradykinin (the GPCR agonist of the G_q_ type) is mediated by CaMKII [61]. In contrast, the active PKC phosphorylates eNOS (Thr-495) and inhibits its activity [154]. This process can be regarded as a negative feedback after endothelial G_q_ receptor activation. The phosphatases may act in an analogous fashion. NO production is modified by various circumstances, such as hypoxia that increases the association of eNOS-HSP90 and PKB [183]. Other EDRFs support (H_2_S) [122] or diminish (CO) the NO production [126]. NO formation decreases after the activation of the endothelial RhoA/ROCK pathway [253]. Last but not the least, NO is not only synthesized de novo. Various products of NO metabolism, such as S-nitrosothiols, nitrites, and nitrates, can serve as reservoirs, capable of re-releasing NO under certain conditions. For example, hemoglobin acts as an oxygen sensor and generates NO from nitrites during hypoxia [96]. Mitochondrial cytochrome c oxidase acts analogously [27]. Via this mechanism, the organism can improve blood flow to hypoxic tissues. On the other hand, hemoglobin also works as an NO scavenger and thus regulates its diffusion into the VSM.

#### Effects of NO

The lifetime of the vascular NO is generally short, depending on the tissue and state and in the vascular lumen it is ~ 2 ms [90, 224]. NO molecules quickly diffuse from the endothelium radially to the VSM cells and activate the NO/sGC/PKG pathway there. The sGC contains a heme moiety that easily binds NO and probably detects even its picomolar levels. The vasodilatory effects of the activated PKG in VSM have already been described (see Sect. Protein kinase G). NO also acts independently of the PKG; the pathway involves the activation of SERCA on the SR by NO-dependent S-glutathionylation via peroxynitrite (and thus depends not only on NO and GSH, but also on the superoxide presence) [2]. In the skeletal muscle, NO directly activates the RyR_1_ channels on the SR by S-nitrosylation [214]; however, the same effect in the VSM cells remains uncertain, since NO was reported to suppress the Ca^2+^ sparks (see Sect. Ca^2+^ sparks and waves in the VSM cells) in the small mesenteric arteries [184]. The vasodilatory effects of NO are supported by a decrease in the plasma membrane potential (becomes more negative) in the endothelial cell which further increases the production of endothelial NO [149] and is transmitted to VSM via MEGJ [196]. In addition, NO may directly open the BK_Ca_ channels and contribute to vasodilatation in some vessels [156]. Simultaneously, NO inhibits endothelin-1 synthesis directly in the endothelium and its downstream cascade [21]. In addition to direct vasoactivity, NO exhibits effects on blood. Its molecule reduces platelet aggregation and the expression of the adhesion molecules on the endothelial surface. Reciprocally, the aggregating platelets release 5-HT and ADP, which increase NO production [234, 235]. Notably, the vasodilatory effects of NO are regarded as beneficial, but excessive NO supplementation was, somewhat surprisingly, found contra-productive in clinical trials [171, 225, 257]. The nitroglycerin-induced endothelial dysfunction, oxidative stress, and nitrate tolerance could result from an imbalance among exogenous NO and endogenous vasoactive factors [205].

**Fig. 4:**
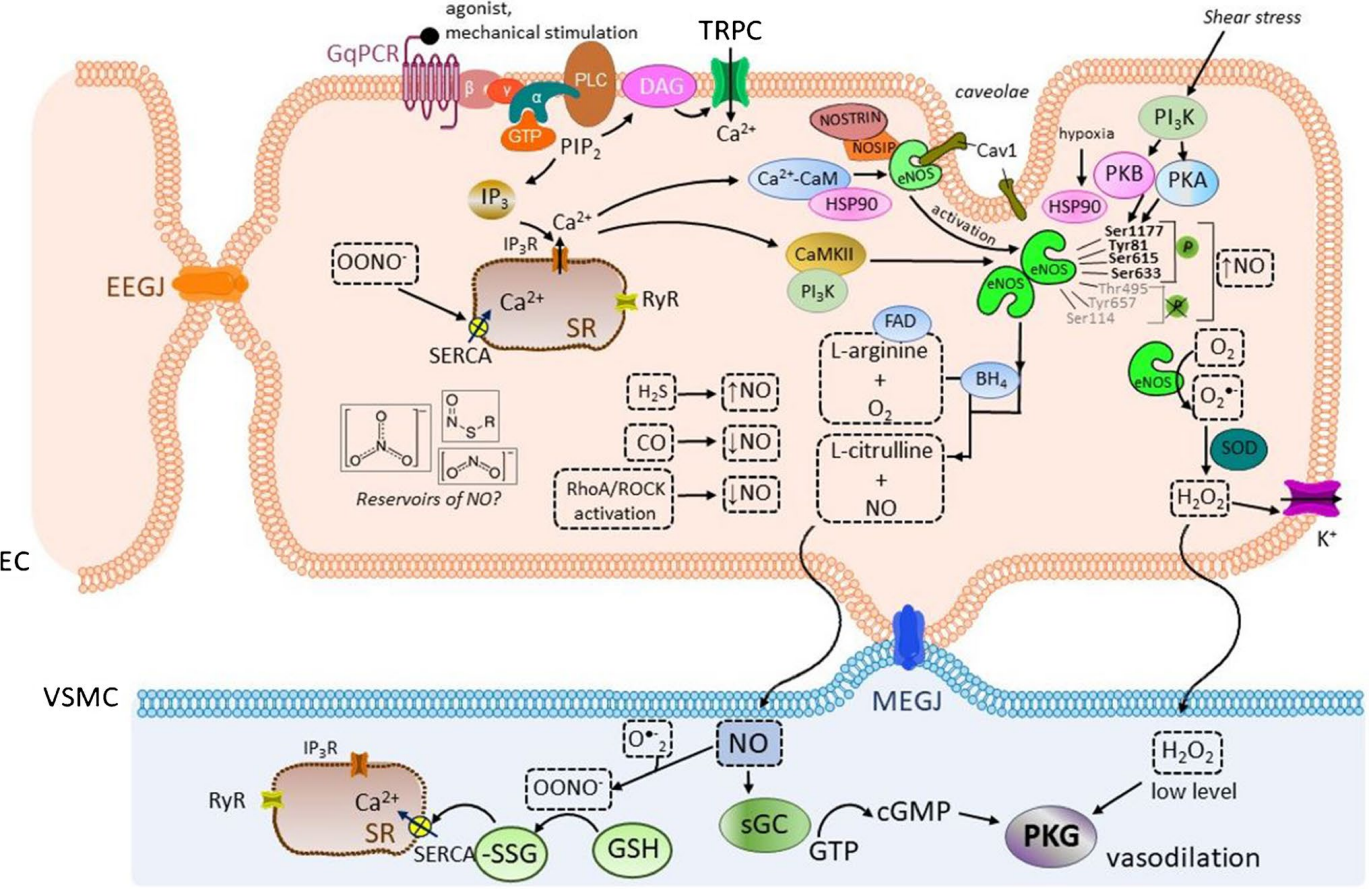
**The role of endothelial nitric oxide synthase (eNOS), nitric oxide (NO), and hydrogen peroxide (H2O2) in the endothelial cells (EC) and the vascular smooth muscle (VSM) cells.** Inactive eNOS is located in the plasma membrane invaginations, *caveolae*, bound to the protein caveolin-1 (Cav1). The binding of an agonist to the G protein-coupled receptor of the G_q_ type (G_q_PCR) on the plasma membrane results in the activation of phospholipase C (PLC) with the ensuing production of diacylglycerol (DAG) and inositol trisphosphate (IP_3_). IP_3_ binds to inositol trisphosphate receptors (IP_3_Rs) on the sarcoplasmic reticulum (SR) and induces the release of calcium from the SR and hence subsequent formation of the calcium-calmodulin (Ca^2+^-CaM) complex. eNOS becomes activated after caveolin-1 displacement by the Ca^2+^-CaM complex. Various cofactors are required for the production of NO, such as tetrahydrobiopterin (BH_4_), flavin adenine dinucleotide (FAD), flavin mononucleotide, and heme. In particular, the BH_4_ concentration is of special importance since BH_4_ enables the dimerization of eNOS and the production of NO. NO and L-citrulline are formed from L-arginine by the action of eNOS in its dimerized form. On the contrary, upon eNOS uncoupling, the eNOS monomers preferentially produce the superoxide anion O_2_^•−^,which can be converted to H_2_O_2_ as another endothelium-derived relaxing factor (EDRF). The activity of eNOS is regulated by several factors, among others the heat shock protein 90 (HSP90) which alters the sensitivity of eNOS to the Ca^2+^-CaM complex. In addition, post-translational modifications of eNOS on tyrosine (Tyr), serine (Ser), and threonine (Thr) sites interfere with NO production. Several kinases participate in the phosphorylation of eNOS, such as the CaMKII and phosphatidylinositol-3-kinase (PI_3_K), protein kinase B (PKB), and protein kinase A (PKA) (also protein kinase C (PKC) and Rho-associated protein kinase (ROCK), not shown in the figure). The shear stress caused by the blood flow is detected by endothelial mechanosensors, giving rise to the activation of PI_3_K, with subsequent activation of PKB and PKA. Then, PKB and PKA phosphorylate eNOS. Other factors, such as hypoxia, increase the association of eNOS-HSP90 and PKB. HSP90 facilitates the displacement of caveolin-1 by calmodulin and potentiates the interaction of eNOS with PKB. In addition, NO production is influenced by factors, such as the eNOS traffic inducer (NOSTRIN) and eNOS interacting protein (NOSIP) which facilitate the translocation of eNOS from *caveolae* to the intracellular region. There are EDRFs that enhance NO production, such as hydrogen sulfide (H_2_S), whereas carbon monoxide (CO) acts as a suppressor. Besides, the inhibition of the endothelial system RhoA/ROCK increases NO production. Interestingly, NO is formed not only de novo, but also as a product of the metabolism of nitrogen compounds, such as S-nitrosothiols, nitrites, and nitrates that can serve as reservoirs, capable of releasing NO under specific conditions. After being formed in the endothelial cells, NO rapidly diffuses to the adjacent VSM cells, where the molecule activates the soluble guanylate cyclase (sCG), with subsequent production of cGMP and protein kinase G (PKG) activation. In the VSM cells, NO can react with O_2_^•−^ to furnish peroxinitrite (OONO^−^), which then activates the sarco/endoplasmic calcium ATPase (SERCA) on the sarcoplasmic reticulum (SR) by glutathionylation. H_2_O_2_ is produced from O_2_^by glutathionylation. H O is produced from−^ under superoxide dismutase (SOD) catalysis. The effects of H_2_O_2_ on EC probably include the activation of the K^+^ channels, and the effects on the VSM are probably dependent on its concentration. While low levels give rise to PKG activation and subsequent vasodilatation, high H_2_O_2_ concentrations act as endothelium-derived constricting factor (EDCF) (not shown in the figure). *Other abbreviations:* cGMP, cyclic guanosine monophosphate; EEGJ, gap junctions between adjacent endothelial cells; GSH, glutathione; GTP, guanosine triphosphate; MEGJ, myoendothelial gap junctions; PIP_2_, phosphatidylinositol bisphosphate; PKA, protein kinase A; PKB, protein kinase B; RyR, ryanodine receptor; SERCA-SSG, sarco/endoplasmic reticulum calcium ATPase S-glutathionylated; _TRP_C, transient receptor potential cation channels

The role of NO in vasodilatation is summarized in Fig. 4.

### H_2_O_2_

#### Production of H_2_O_2_

Endothelial H_2_O_2_ synthesis (mainly) from the superoxide anion O_2_^•−^is catalyzed by superoxide dismutase (SOD). The superoxide radical-anion is produced by many enzymatic systems, including NADPH oxidase, xanthine oxidase, NOS, mitochondrial respiratory chain, and also as a by-product of epoxyeicosatrienoic acid synthesis (see Sect. Epoxyeicosatrienoic acids). In the vascular system, the NADPH oxidases are the main O_2_^•−^generators [39]. NOX complexes produce ROS using NAPDH and O_2_ as substrates. Various NOX isoforms were found in humans (5 isoforms) and rodents (4 isoforms) [13, 39, 124, 159]. Superoxide produced by eNOS is one of the important precursors of H_2_O_2_ in the endothelium (see Fig. 4). However, there are differences among species, specific arterial beds, and influence of factors, such as development, age, and general health state [133, 146–148, 204, 205]. The eNOS contains oxygenase and reductase domains, where superoxide is produced. In the latter case, the production is independent of the Ca^2+^-CaM complex, and unmodifiable by eNOS inhibitors, such as L-NAME. Superoxide is physiologically produced together with NO, and subsequently gives rise to the formation of H_2_O_2_, which acts as one of the EDH factors (see Sect. Endothelium-dependent hyperpolarization). Upon eNOS uncoupling to monomers, superoxide production prevails, and shifts the NO/superoxide ratio. The relative excess of the superoxide is not only reflected in an increased amount of H_2_O_2_, but also in the conversion to other metabolites. Among them, the highly reactive peroxynitrite ONOO^−^ arises from the reaction with the remaining NO, whose availability is diminished this way. The important cofactor BH_4_ is among the molecules oxidized by the peroxynitrite; its deficiency results in further monomerization of eNOS and the decrease of NO production.

#### Effects of H_2_O_2_

Cytosolic concentration of H_2_O_2_ is the key factor that determines its vascular effects. As an EDH factor, its level is quite low, ranging approximately from units to low tens of µM concentrations [205, 249]. In the VSM, H_2_O_2_ molecules act principally through the PKG activation, which leads to vasodilatation (see Sect. Protein kinase G) [47, 258]. At higher concentrations, hydrogen peroxide acts as EDCF and mediates vasoconstriction, probably via elevated levels of contractile factors, such as thromboxane TxA_2_, excessive superoxide production, and elevated [Ca^2+^]_c_ in VSM [67]. The H_2_O_2_-derived ROS have been found to upregulate connexins Cx40 and Cx45 in human cardiomyocytes [121] (the Cx40 may participate in endothelium dependent vasodilatation conducted along the vessel [208]). The involvement of H_2_O_2_ and gap junctions in endothelium-derived hyperpolarizing response to bradykinin was observed in omental arteries and veins, isolated from pregnant women [84].

Even though H_2_O_2_ molecules are formed in vessels of all diameters, its role significantly increases in microcirculation. The dominance of H_2_O_2_ over NO may be at least partially mediated by the less intense NO production in small vessels due to augmented eNOS inactivation by caveolin-1 [194]. Importantly, NO and H_2_O_2_ do not compete, but rather complement each other as vasodilators. The key role H_2_O_2_ in microcirculation is supported by the finding that a long-term antioxidant therapy does not improve patient mortality [17]. However, the role of NO and H_2_O_2_ cannot be generalized. The controlling mechanisms are highly heterogenous among arteries and even within one artery (a proximo-distal manner) and influenced by a plethora of factors (development, aging, general health, and disease). The endothelium is considered as the principal source of H_2_O_2_, but it is also synthesized directly in the VSM cells. Interestingly, H_2_O_2_ is among the vasodilatory substances produced by the brown adipose tissue, and this source was reported to induce the PKG-1_α_ activation [65, 93]. Surprisingly, the extracellular production of superoxide can result in redox modifications of intracellular domains including eNOS. This effect could be mediated by the conversion of O_2_^.−^ to H_2_O_2_ which has higher stability as well as the ability to cross biological membranes [22].

### Vascular gasotransmitters

Gasotransmitters are small gaseous molecules, generated in cells under both physiological and pathological conditions. They are dissolved in biological fluids and freely pass through the plasma membrane and intracellular organelle membranes. They interact with plenty of targets and influence various biological pathways. The best known vascular gasotransmitters are H_2_S, CO, and, in principle, also NO, but, for historical reasons, it is usually dealt with separately.

#### H_2_S

H_2_S is generated from *L*-cysteine. In the vascular system, its production is catalyzed mainly by cytosolic cystathionine γ-lyase. Other ways of production mediated by cystathionine-β-synthase and cysteine aminotransferase/3-mercaptopyruvate sulfurtransferase are minor. It is also formed in PVAT. The production of H_2_S is stimulated by the Ca^2+^-CaM complex, hypoxia, NO, ROS, and shear stress, and decreases with age, obesity, hypertension, and diabetes. Hydrogen sulfide participates in signal transduction, mainly through persulfidation of cysteine residues (-SSH) in important biomolecules. Among other functions, the molecule exhibits vasodilatory action. Effects of H_2_S in the VSM cells are complex. Hydrogen sulfide (1) activates the potassium channels (the BK_Ca_, K_v_7.4, and K_ATP_) leading to a decrease in membrane potential; (2) inhibits the IP_3_R receptors and decreases [Ca^2+^]_c_; (3) decreases the intracellular pH via Cl^−^/HCO_3_ exchanger; (4) increases the cGMP levels, probably through the PDE inhibition; and (5) reacts with NO to furnish nitrosothiol and thus decreases NO availability. In the endothelium, H_2_S activates the small (SK_Ca_) and intermediate (IK_Ca_) calcium-activated K^+^ channels which leads to a decrease in membrane potential. Importance of H_2_S increases with decreasing diameter of the vessel [108, 246, 250]. H_2_S donors may be therapeutically promising, and many structural types are studied for this purpose (sulfide salts, allicin derivatives, cysteine derivatives) [260].

#### CO

Carbon monoxide physiologically arises during heme degradation, catalyzed by hemoxidase. Its production is enhanced by various stimuli, such as hypoxia and hypotension, and the increase is based on hemoxidase posttranslational modifications with the participation of the Ca^2+^-CaM complex. In the vessels, CO exhibits vasodilatory effects, including (1) support of the frequency and amplitude of Ca^2+^ sparks; and (2) the sparks coupling with the BK_Ca_ channels. Accordingly, the hemoxidase is colocalized with the BK_Ca_ channels. CO (3) activates the sGC; however, it is a weaker activator than NO. Similar to the H_2_S donors, CO-releasing molecules (CORMs) are searched for, with organometallic compounds, oxalates, aldehydes, and boranocarboxylates being tested among others [126, 132, 175].

#### Interplay of gasotransmitters

The gasotransmitters mutually influence the formation and, likely, also the action of each other. NO stimulates both cystathionine γ-lyase and hemoxidase. In contrast, CO inhibits both the cystathionine γ-lyase and eNOS, while H_2_S inhibits the hemoxidase, but stimulates NO production. The latter process is executed via the S-sulfhydration of eNOS that prevents the enzyme monomerization, through the eNOS phosphorylation by PKB, and through Ca^2+^ released from the SR. NO and H_2_S might also interact together and form a yet uncovered vasorelaxation mediator [246].

**Fig. 5:**
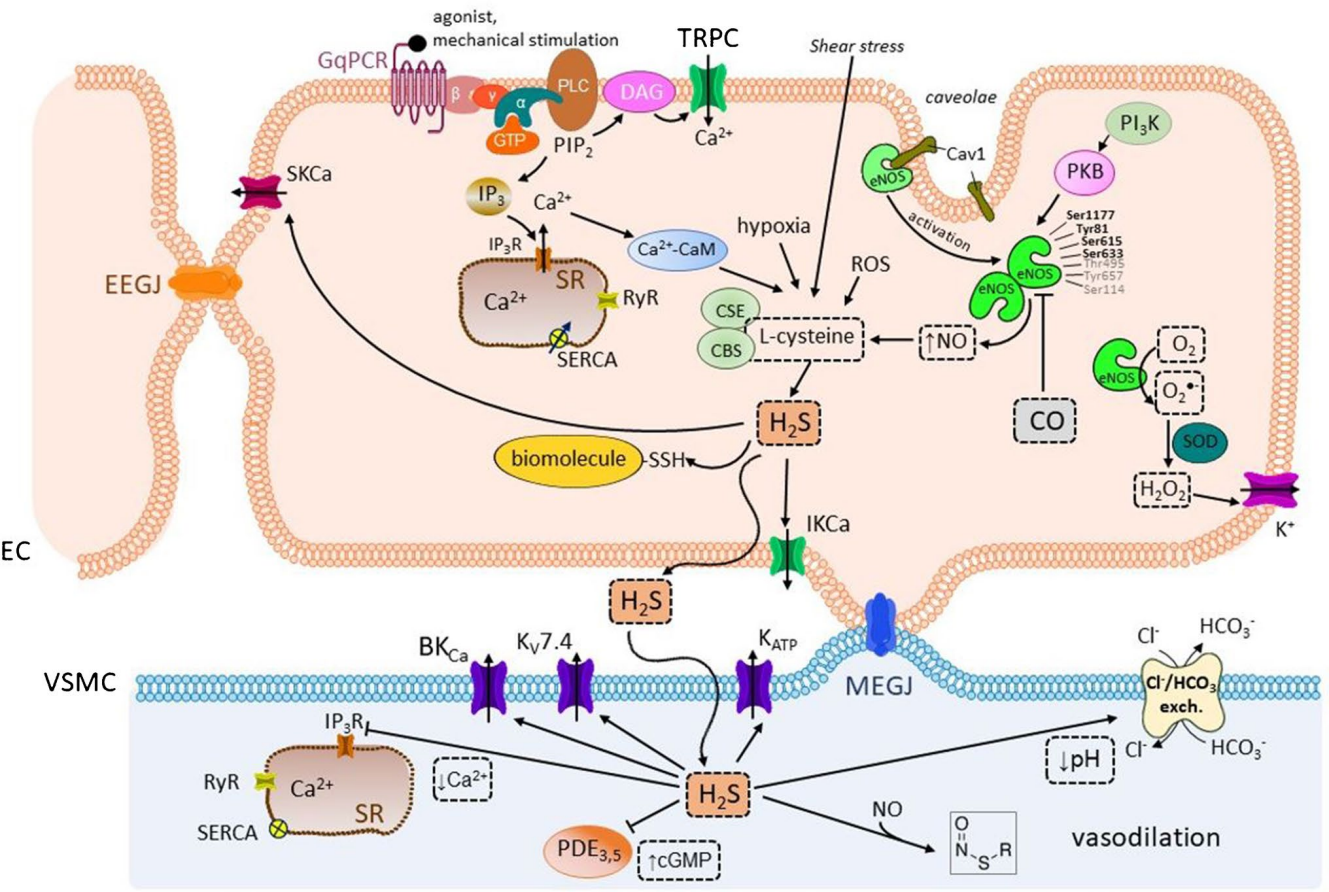
**The role of the gasotransmitters hydrogen sulfide (H2S) and carbon monoxide (CO) in the endothelial cells (EC) and the vascular smooth muscle (VSM) cells**. H_2_S is formed from L-cysteine. The formation is catalyzed mainly by the cytosolic cystathionine γ-lyase (CSE), while minor pathways involve cystathionine-β-synthase (CBS) and cysteine aminotransferase/3-mercaptopyruvate sulfurtransferase (3-MST).Various stimuli increase H_2_S production, such as the calcium-calmodulin complex (Ca^2+^-CaM), hypoxia, nitric oxide (NO), reactive oxygen species (ROS), and shear stress. After being produced, H_2_S participates in signal transduction, mainly through persulfidation of the cysteine residues (-SSH) in physiologically important biomolecules. In EC, H_2_S activates the small-conductance and intermediate-conductance calcium-activated K^+^ channels (SK_Ca_ and IK_Ca_, respectively), which decreases membrane potential. In addition, H_2_S diffuses into the adjacent VSM cells where the substance (1) activates the potassium channels (large conductance calcium-activated K^+^ (BK_Ca_), voltage-gated K^+^ (K_V_7.4), and ATP-dependent K^+^ (K_ATP_) channels) thus contributing to the membrane potential decrease (becomes more negative); (2) inhibits the inositol trisphosphate receptors (IP_3_R) on the sarcoplasmic reticulum (SR) leading to a decrease in the cytosolic calcium concentration [Ca^2+^]_c_; (3) decreases intracellular pH via the Cl^−^/HCO_3_^−^ exchanger; (4) increases the cGMP levels, probably through phosphodiesterase (PDE) inhibition; and (5) reacts with NO to give nitrosothiol, which decreases NO bioavailability. The overall net effect of all these pathways is VSM relaxation. In EC, CO inhibits endothelial nitric oxide synthase (eNOS). *Other abbreviations:* Ca^2+^-CaM, complex calcium-calmodulin; Ca_v_1, caveolin-1; cGMP, cyclic guanosine monophosphate; DAG, diacylglycerol; EEGJ, gap junctions between adjacent endothelial cells; G_q_PCR, G protein-coupled receptor of G_q_ type; GTP, guanosine triphosphate; IP_3_, inositol trisphosphate; MEGJ, myoendothelial gap junctions; PI_3_K, phosphatidylinositol-3-kinase; PIP_2_, phosphatidylinositol bisphosphate; PKB, protein kinase B; PLC, phospholipase C; ROS, reactive oxygen species; RyR, ryanodine receptors; SERCA, sarco/endoplasmic reticulum calcium ATPase; SOD, superoxide dismutase; TRPC, transient receptor potential cation channel

The role of gasotransmitters in vasodilatation is summarized in Fig. 5.

### Endothelial derivatives of arachidonic acid

Arachidonic acid is released from the plasma membrane by phospholipase A_2_ (PLA_2_). There are three subtypes of PLA_2_, secreted sPLA_2_, cytosolic cPLA_2_, and calcium-independent iPLA_2_. Among them, the cPLA_2_ is the most important for the arachidonic acid release in the vascular system. PLA_2_ is activated by an increase in endothelial [Ca^2+^]_c_, brought about by agonists on the endothelial GPCR of the G_q_ type [242] as well as in response to the blood-flow induced shear friction [59]. Notably, the same stimuli increase NO production, which inhibits further EDH-dependent vasodilatation (see Sect. Endothelium-dependent hyperpolarization) and provides a negative feedback [168]. The released arachidonic acid is metabolized by three principal pathways: by cyclooxygenase (COX-1 and 2), cytochrome P450 (CYP), and lipoxygenase (LOX) [157]. The COXs yield prostaglandin PGH_2_ which is further converted to PGI_2_ (prostacyclin), PGE_2_, PGF_2α_, and thromboxane TxA_2_. The CYP are heme-containing enzymes producing four epoxyeicosatrienoic acids (EETs) and hydroxyeicosatetraenoic acids (HETEs), such as 20-HETE. LOX metabolizes arachidonic acid into leukotrienes (LTs), and various HETEs are also synthesized. The formation of individual metabolites depends on the cell type and available enzymes. The resultant effect is further determined by the expression of the receptors in individual tissues. Importantly, the metabolites of the same enzymatic pathway can have opposite vascular effects. Thus, the COX product PGI_2_ produces vasodilatation, while thromboxane TxA_2_ is vasoconstrictive. The same is true for the EETs and 20-HETE. Depending on the conditions, the effects can be variable. Thus, the PGE_2_ is usually a vasodilatory compound; this prostanoid, however, displays contractile effects upon central administration to the cerebral ventricles [252]. The regulation of the blood flow by the products of arachidonic acid metabolism is based on the interplay among them, with the ratio between PGI_2_ and TxA_2_ having probably the key role. Among EDRFs, the PGI_2_ and EETs are of the highest importance.

#### Prostaglandin PGI_2_

In the vascular system, the PGI_2_ production has been associated with the endothelial COX-2; however, COX-1 may predominate under physiological conditions. Accordingly, the COX-1 and PGI_2_ synthase are co-localized [110]. The PGI_2_ synthase is by far the most abundant prostanoid synthase expressed in the endothelial cells (85.9% in normotensive rats and 93.7% in spontaneously hypertensive rats SHR, both 36 weeks old) [220]. Consequently, the PGI_2_ is the main COX product formed in the endothelium. In females, its production is stimulated by estrogens [211], in particular 17β-estradiol [6]. Following its release from the endothelium, the PGI_2_ stimulates the IP_1_ receptors on the VSM cells (GPCR of the G_s_-type) and activates the AC/cAMP/PKA and AC/cAMP/EPAC cascades described above (see Sect. Protein kinase A). Analogous to NO, the PGI_2_ inhibits the platelet aggregation [210]. Importantly, the PGI_2_ vasodilatation decreases with aging. Accordingly, the expressions of COX-1, COX-2, and TxA_2_ synthases but not that of the PGI_2_ synthase increased in the rats of 36 and 72 weeks of age [98, 220].

#### Epoxyeicosatrienoic acids

The EETs are formed as a result of the catalytic activity of the CYP epoxigenases 2 C8/9. Their molecules induce the NO-independent vasodilatation in multiple ways. At micromolar concentrations, they activate the endothelial and smooth muscle K_Ca_ channels and cause a decrease in the plasma membrane potential [178]. The acids are also agonists of the GPR40 receptor, which is coupled with both G_q_ and G_s_ proteins. The EETs probably interact with some other sites as well [177]; the TRPV_4_ and TRPC_1_ channels are among the candidates. Since they form a complex with the BK_Ca_ channels on the VSM, these channels may become activated in this way [141, 239]. Two possible mechanisms of the EETs-mediated vasodilatation have been described: (1) on the endothelial cells, they may activate the GPCR of the G_q_ type with [Ca^2+^]_c_ increase, which results in the IK_Ca_ and SK_Ca_ channel activation and re-/hyperpolarization. The membrane potential is transferred to the adjacent VSM cells through the activation of the Na^+^/K^+^ ATPase and K_ir_ channels opening and via the MEGJ; or (2) the GPCR of the G_s_ type may be activated directly on the VSM cells with the opening of the BK_Ca_, K_v_, and subsequently of K_ir_ channels. Activation of the BK_Ca_ channels may be mediated by CO, which is produced after the activation of heme oxygenase [104, 192]. Both mechanisms may operate simultaneously, depending on the type of the vessel and species [23, 71, 228, 254, 255]. Furthermore, the role of individual EETs may not be identical. 11,12-EET is generated during hypoxia and targets the endothelial TRPC_6_ [112], whereas the action of the 5,6-EET is associated with mechano- and osmotic stimulation and activation of the TRPV_4_ channels [15, 239]. The EETs are metabolized by the soluble epoxide hydrolase (sEH) to dihydroxyeicosatetraenoic acids (DHETEs), whose biological activities are not fully understood yet; however, it is likely that they modify the endothelial Ca^2+^ levels [83].

#### Products of LOXs

The endothelial cells contain various lipoxygenases. 5-LOX produces leukotrienes with concomitant collaboration of the 5-LOX-activating protein (FLAP). 12/15-LOX, 12-LOX, and 15-LOX produce a number of metabolites, among them 12(*S*)-hydroxyeicosatrienoic acid (12-HETE), 15-HETE, and 11,12,15-trihydroxyeicosatrienoic acid (11,12,15-THETA). The 15-LOX pathway represents the first known example of inducible endothelium-derived hyperpolarizing factor [24]. Notably, 12-LOX is also present in the blood platelets. In general, the LOX products are vasoactive and can both dilate and constrict arteries, depending on the vascular bed and species. This vasodilatation includes the activation of the BK_Ca_ channels [57].

**Fig. 6:**
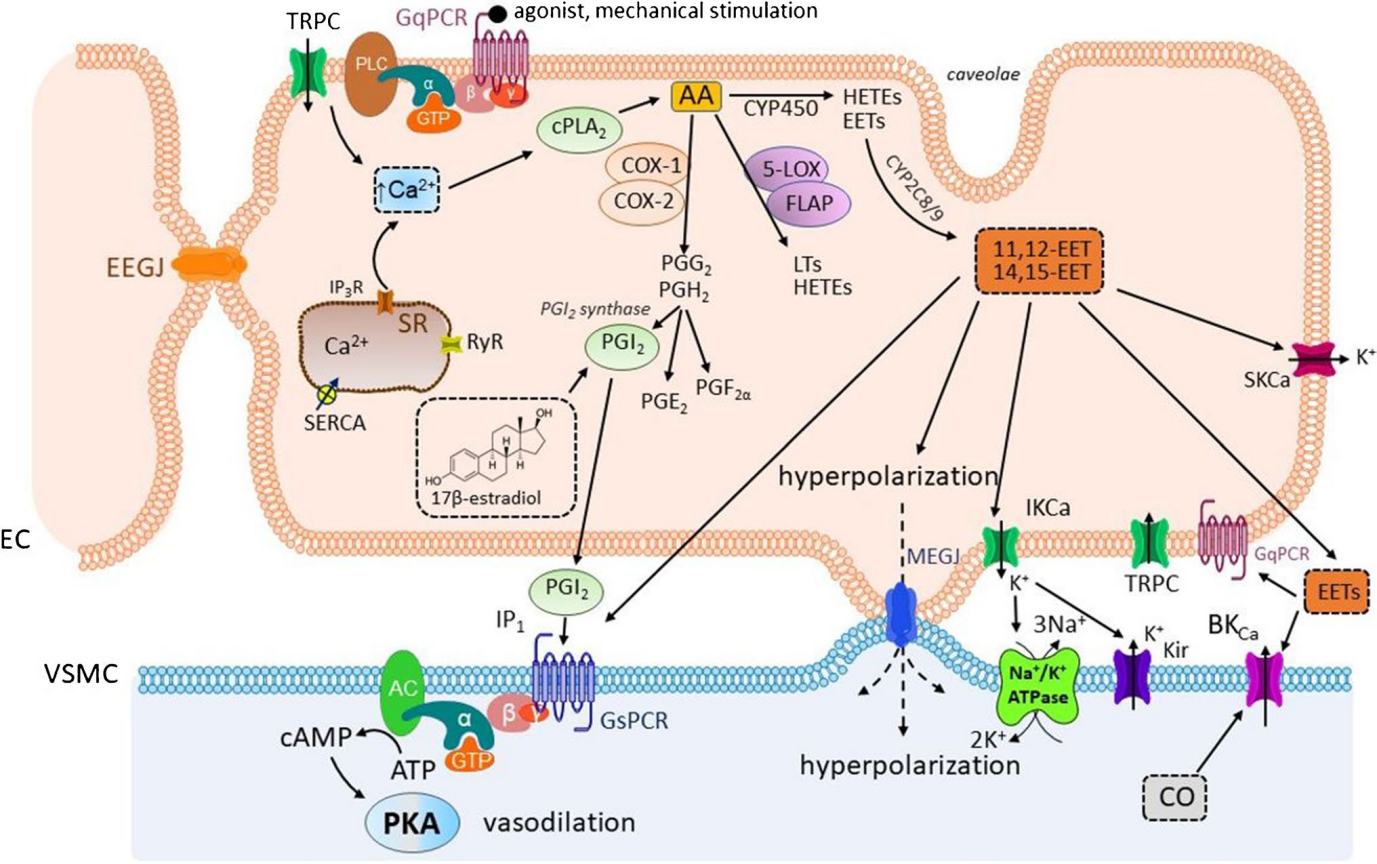
**The role of derivatives of arachidonic acid (AA) produced in the endothelial cells (EC) in the vascular smooth muscle (VSM) cell relaxation**. The binding of an agonist to the G protein-coupled receptor of the G_q_ type (G_q_PCR) causes an increase in the cytosolic calcium concentration [Ca^2+^]_c_ on the ECs which, in turn, activates phospholipase A_2_ (PLA_2_). By the action of PLA_2_, arachidonic acid (AA) is released from the membranes and afterwards metabolized by cyclooxygenases (COX-1 and COX-2), cytochrome P450 (CYP), and lipoxygenase (LOX). The COX yield prostaglandin H_2_ (PGH_2_) which can be further converted into prostaglandins PGI_2_ (prostacyclin), PGE_2_, and PGF_2α_. The CYP are membrane and heme-containing enzymes producing four epoxyeicosatrienoic acids (EETs) and hydroxyeicosatrienoic acids (HETEs). The 5-lipoxygenase (5-LOX) and 5-lipoxygenase activating protein (FLAP) metabolizes arachidonic acid into leukotrienes (LTs) and various hydroxyeicosatrienoic acids. The metabolites of one enzymatic pathway can have opposite effects on the vascular system. For instance, the COX product prostacyclin (PGI_2_) is vasodilatory, while thromboxane (TxA_2_), produced by the platelets, is vasoconstrictive. PGE_2_ is generally a vasodilatory prostanoid, but the substance also can, under certain conditions, act as vasoconstrictive. It is worthy to mention that the regulation of the blood flow by the metabolites of AA is based on the interplay among them, with the ratio between PGI_2_ and TxA_2_ being the most important. The PGI_2_ synthase is the most abundant prostanoid synthase expressed in ECs and, consequently, PGI_2_ is the main COX product formed in the endothelium. Endothelial production of PGI_2_ is stimulated by estrogens (e.g., 17β-estradiol). After being released from the endothelium, PGI_2_ stimulates its IP_1_ receptor on the VSM. This G protein-coupled receptor of the G_s_ type (G_s_PCR) activates adenylate cyclase (AC) with subsequent production of cAMP and protein kinase A (PKA) activation. EETs are formed by CYP epoxygenases 2 C8/9. EETs produce vasodilatation by activation of the G protein-coupled receptor of the G_q_ type/phospholipase C/inositol trisphosphate + diacylglycerol/protein kinase C (G_q_ type/PLC/IP_3_ + DAG/PKC) pathway on EC. This pathway involves the transient receptor potential cation channels (TRPC_1_, TRPC_3_, and TRPC_6_), which enable an influx of [Ca^2+^]_c_. Subsequent activation of the small-conductance and intermediate-conductance calcium-activated K^+^ channels (SK_Ca_ and IK_Ca_, respectively) results in the decrease of membrane potential (becomes more negative), which is transferred to the VSM through the myoendothelial gap junctions (MEGJ) and via the activation of Na^+^/K^+^ ATPase and inward-rectifier K^+^ (K_ir_) channels. Another possible mechanism is the activation of the G protein-coupled receptor of the G_s_ type/adenylate cyclase/cyclic adenosine monophosphate/protein kinase A (G_s_PCR/AC/cAMP/PKA) pathway directly on the VSM with subsequent opening of the large conductance calcium-activated K^+^ channels (BK_Ca_), voltage-gated K^+^ channels (K_v_), and inward-rectifier (K_ir_) channels. *Other abbreviations:* ATP, adenosine triphosphate; cAMP, cyclic adenosine monophosphate; CO, carbon monoxide; EEGJ, gap junctions between adjacent endothelial cells; GTP, guanosine triphosphate; IP_3_R, inositol trisphosphate receptors; MEGJ, myoendothelial gap junctions; RyR, ryanodine receptors; SERCA, sarco/endoplasmic reticulum calcium ATPase; SR, sarcoplasmic reticulum.

The role of endothelial derivatives of arachidonic acid in vasodilatation is summarized in Fig. 6.

### Other endogenous vasodilatory substances

The natriuretic peptide C (CNP) is formed and stored in the endothelium. The regulation of its expression is complex. It is enhanced by, e.g., shear stress [172], the transforming growth factor β, or the tumor necrosis factor α, and suppressed by the vascular endothelial growth factor (VEGF) [202]. The CNP vasodilatation is based on the activation of the membrane pGC with the initiation of the cGMP/PKG pathway [30].

Insulin produces vasodilatation which is mediated through the PKB activation of eNOS [1, 162] but not by opening of the K_Ca_ and K_ATP_ channels [1]. Accordingly, insulin resistance is associated with a decreased bioavailability of NO and endothelial dysfunction [238].

Ghrelin is another vasoactive substance capable of dilating the small arteries via the mediacy of NO and possibly also NO-independently. The substance exhibits indirect vascular effects through the stimulation of diuresis and possibly through the autonomic system [145, 179, 180].

Vasodilatory effects were reported for the active form of vitamin D, paricalcitol, in the chronic kidney disease patients [263]. Similarly, adiponectin, an important adipocytokine secreted by the adipocytes, brings about NO-dependent vasodilatation of the retinal arterioles, which is partially dependent on AMPK activity [174]. The VEGF is not only a key factor during angiogenesis, but it also modulates NO production [247]. The therapy with VEGF inhibitors is associated with the risk of hypertension and embolism [158, 181]. The endothelium-dependent vasodilatation may be supported by a number of natural substances, such as polyphenols including flavonoids [42, 97, 153, 164, 170, 182] and phytoestrogens. Notably, the same is true for the selective estrogen receptor modulators (SERMs) [128, 226, 244].

### Endothelium-dependent hyperpolarization

The hyperpolarization of the VSM plasma membrane leads to vasodilatation. If this hyperpolarization originates from the endothelium, it is referred to as EDH. Hyperpolarization is transmitted to the VSM in two main ways: (1) by K^+^ ions effluxed by the endothelial Ca^2+^- activated potassium channels (IK_Ca_ and SK_Ca_); and (2) by MEGJ.

#### Triggering of EDH

The EDH can be triggered by various mechanisms. (1) Ca^2+^ ions are spontaneously released from the endothelial SR through the IP_3_R channels, and activate the K_Ca_ channels in their proximity [125]. Similarly, (2) the spontaneous extracellular Ca^2+^ sparklets are secured by certain TRP channels [12, 50, 176] and their probability increases with the decrease in intraluminal blood pressure below ~ 60 mmHg [12, 206]. (3) Increased [Ca^2+^]_c_ in the VSM cells during vasoconstriction leads to a rise in [Ca^2+^]_c_ in the adjacent endothelial cells as well. This “negative feedback” is not fully understood and could be mediated by IP_3_ or by Ca^2+^ itself passing from the VSM cells into the adjacent endothelial cells through the MEGJ. The [Ca^2+^]_c_ increase can also be triggered for example by agonist binding the endothelial GPCR of G_q_ type. Additionally, the vasoconstrictors can activate the TRPV_4_ channels in myoendothelial projections which results in Ca^2+^ sparklets and endothelial K_Ca_ channel activation (reported for mouse mesenteric arteries) [95]. Last but not the least, (4) increase in endothelial [Ca^2+^]_c_ can be mediated by the activation of several endothelial shear stress responsive ion channels [142, 152]. Regarding the shear stress, new details have recently emerged. The endothelial cells are constantly exposed to the force of blood flow and rolling erythrocytes. In detection of the mechanical stimulation, the membranal channels play an important role. Among them, the K_ir_ channels seem to be of special interest. The K_ir_ channels are classically involved in maintaining resting membrane potential. In the vessels, the K_ir_2.x channel subtype is the most abundant. Endothelial K_ir_2.1 channels are shear-sensitive and may represent the major contributor of flow induced dilatation [3, 173, 199]. The cytoskeletal and scaffolding proteins also contribute to mechanosensing. These proteins interact and modify the signaling microdomains at the cell membrane. Caveolin-1, a protein which participates in the formation and stabilization of *caveoleae*, negatively regulates the K_ir_2 channel activity [85, 199]. Actin, the most abundant cytoskeletal protein, can switch from polymerized to depolymerized states according to external mechanical stimuli [29]. Dystrophin, a sub-membrane cytoskeletal protein, interacts with various proteins including syntrophins forming a dystrophin-associated protein complex (DAPC) which anchors the actin cytoskeleton to the extracellular matrix [37, 53, 199]. The syntrophins were shown to connect K_ir_2 channels directly to DAPC and cytoskeleton [127, 199]. Additionally, the functionality of K_ir_ channels is influenced by local lipid microenvironment in the cell membrane, in particular by the content of phosphatidylinositol 4,5-bisphosphate (PIP_2_) and cholesterol [199]. While the PIP_2_ stabilizes the K_ir_ channels in the active (open) state [86], cholesterol exhibits an opposite effect [129]. Thus, the endothelial dysfunction during hypercholesterolemia may be at least partially explained by K_ir_2.1 channel inhibition [4, 199].

The endothelial K_ir_2.x channels also participate (together with other mediators) in a quick increase in the local cerebral blood flow due to neuronal activity (neurovascular coupling) in the brain arteries [31, 58, 161]. The neuronal action potential is accompanied by an increase in the perivascular K^+^ concentration [92]. This perivascular K^+^ elevation leads to endothelial K_ir_ channel activation in the capillaries, membrane hyperpolarization, and vasodilatation [10, 49, 161] and subsequent retrograde vasodilatory signal propagation [136]. The neurovascular coupling is complex and includes not only the neurons and endothelial cells, but also the astrocytes [99] and pericytes [81] in the vicinity. Moreover, the active neurons could release not only K^+^ but also a mediator, possibly PGE_2_ [138]. If it is so, activation of PGE_2_ receptors and the downstream cascade follow with the subsequent IP3R-mediated signaling and increase in endothelial [Ca^2+^]_c_.

#### K^+^ ions and MEGJ in endothelium-dependent hyperpolarization

The K^+^ ions are effluxed by the endothelial IK_Ca_ and SK_Ca_ channels [70] which are activated by an increase in endothelial [Ca^2+^]_c_. Increase in extracellular K^+^ ion concentration in the proximity of the neighboring VSM cell activates the Na^+^/K^+^ ATPases together with the K_ir_ channels (amplification of the IK_Ca_- and SK_Ca_-initiated hyperpolarization), and subsequently results in a decrease of membrane potential (more negative). The calcium-sensing receptors (CaSR) might secure the activity of the endothelial K_Ca_ channels only within a certain range of [Ca^2+^]_c_, while Ca^2+^ concentration above a certain threshold (~ 1 mM) causes their inhibition [25]. It is noteworthy that a pronounced increase in extracellular K^+^ (above ~ 20 mM) induces vasoconstriction. The participation of CaSR in EDH is not generally accepted and may play a role only in some vascular beds and states. In the rabbit mesenteric arteries, this vasodilatation was endothelium-dependent with increased NO production and IK_Ca_ channel activation [78]. In the male rat mesenteric arteries, the CaSR on the perivascular sensory nerves mediated vasodilatation, which involved both endothelium-dependent and endothelium-independent mechanisms, and a role of the calcitonin gene-related peptide and neutrokinin 1 receptors was reported [26]. Importantly, in addition to the endothelial IK_Ca_ and SK_Ca_ channel activation and subsequent hyperpolarization, the increase in endothelial [Ca^2+^]_c_ will also trigger production and release of vasodilatory factors such as NO, H_2_O_2_, PGI_2_, and EETs, which will also mediate vasodilatation of the adjacent SMC.

The EDH is also transmitted directly by the gap junctions with bidirectional transfer of the membrane potential (and possibly of small molecules and ions). The MEGJ are composed of connexin proteins forming a hexameric hemichannel. Among the connexins, the Cx40 could play a crucial role in EDH conduction longitudinally along the vessel wall [208]. The impact of K^+^ ions and the gap junctions varies according to the vessel type. As the number of MEJGs increases with decreasing arterial diameter [197], the contribution of MEGJ to EHD in small vessels is probably more significant. The role of mediator (Ca^2+^, IP_3_) transfer through the gap junctions between adjacent endothelial cells (EEGJs) is not clear, but there are some studies supporting this [68, 95, 227].

#### Radial and axial transmission of EDH

The endothelial K_Ca_ channels and the gap junctions that are located towards the neighboring VSM cells allow radial propagation of EDH and induction of vasodilatation. The K_Ca_ channels and the gap junctions which are located towards the neighboring endothelial cells are equally important, since they mediate the axial propagation of the initially local vasodilatation along the vessel [44, 102, 103]. There are two types of the endothelial K_Ca_ channels with different localization [45, 198]. The localization is highly variable within and between beds and states. The IK_Ca_ channels are abundant at some MEGJ, and are also located over the surface of the endothelium. The SK_Ca_ are concentrated towards the adjacent endothelial cells, but these channels are also over the surface of the endothelium. Different roles of both channel types are supported by their different status in hypertension, where the function/expression of the IK_Ca_ channels is enhanced [73, 198] while that of the SK_Ca_ channels suppressed [118, 201, 241]. In certain vessels (observed ex vivo in rat middle cerebral artery), the activation of the SK_Ca_ channels may be important for NO-dependent vasodilatation, whereas that of the IK_Ca_ channels for the NO-independent mode (in the absence of NO, IK_Ca_ underpined endothelium-dependent hyperpolarization and relaxation) [150].

For EDH, the co-localization of all the important structures (the K_Ca_ channels, CaSR, Na^+^/K^+^ ATPase, K_ir_ channels, IP_3_R) in the proximity of the gap junctions is optimal. At least partially, they could be anchored to the plasma membrane by the AKAP150 as the scaffolding protein. The TRPV_4_ channels are present in the same cluster [212]. These channels not only secure the spontaneous Ca^2+^ sparklets but can also be activated by the agonist of GPCR of the G_q_ type with the activation of the IP_3_ + DAG/PKC/AKAP/TRPV_4_ pathway. Besides, the TRPV_4_ channels probably also work as the shear stress sensors [228]. In this way, the endothelium at least partially mediates the physiological autoregulatory response to pressure changes [87]. The activity of the endothelial TRPV_4_ channels is impaired under cardiovascular diseases [33]. Surprisingly, their upregulation was reported in the hypertension induced by high sodium intake [66], which could be explained by a compensatory mechanism. Other endothelial TRP channels also participate in endothelial vasodilatation [50]. For example, the TRPA_1_ channels are involved in the sensing of extremely wide variety of stimuli (chemical, thermal, and mechanical, including stretch sensitivity) [219, 228]. Accordingly, the endothelium-dependent artery dilatation mediated by the TRPA_1_ and K_Ca_ channels was reported on isolated pressurized rat cerebral arteries [51]. In addition, various TRP channels participate in endothelial the store-operated extracellular Ca^2+^ entry (SOCE) and thus indirectly influence EDH; however, the endothelial SOCE can also be independent of them [223]. The chloride channels are important for the physiological EDH as well. Upon activation, these channels give rise to depolarization, and their upregulation counteracts the EDH [76, 140].

**Fig. 7:**
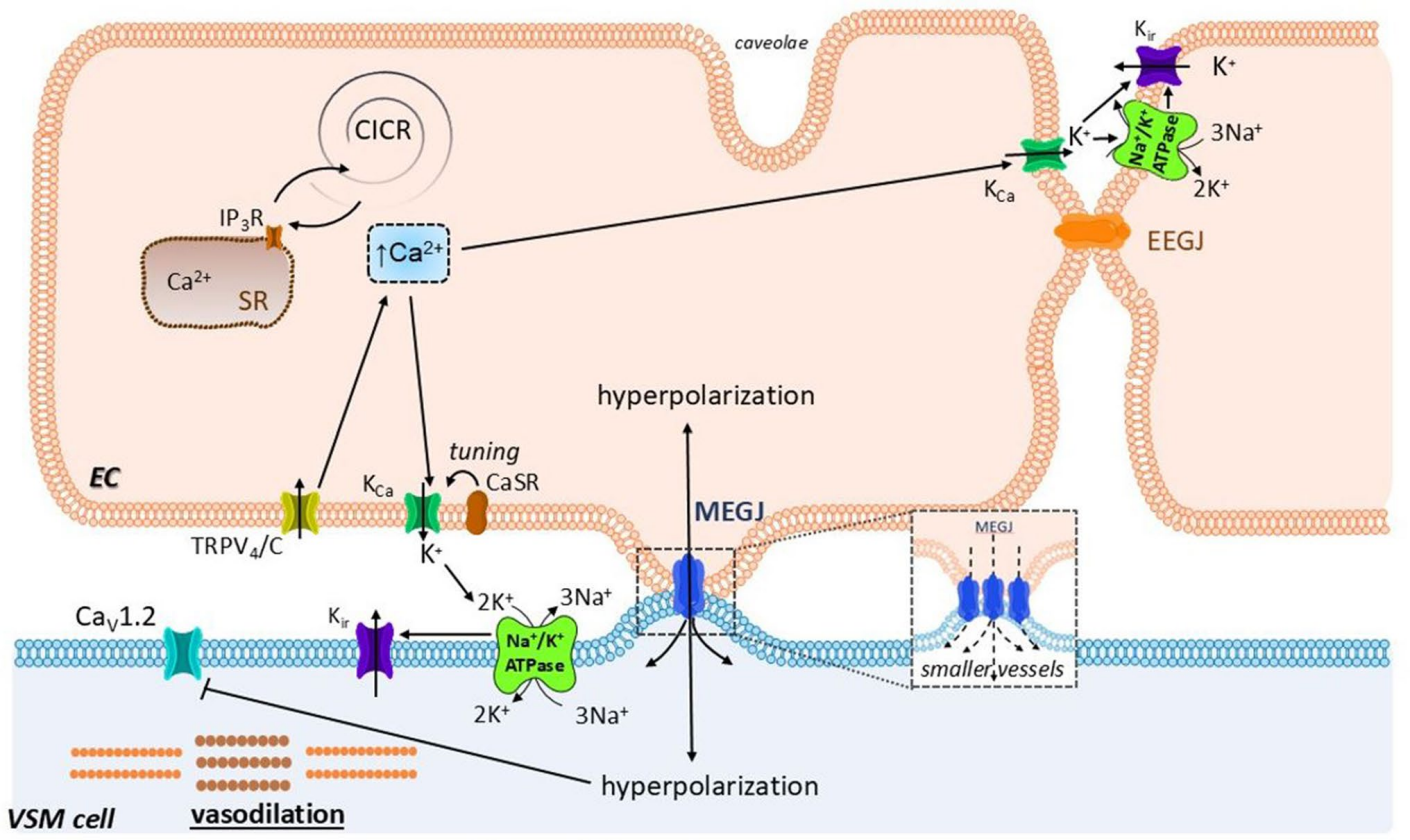
**The role of the endothelium-dependent hyperpolarization (EDH) in the relaxation of the vascular smooth muscle (VSM) cells**. A decrease in the membrane potential of endothelial cells (EC) gives rise to vasodilatation. The conduction of the hyperpolarization from ECs to VSM cells is mediated by the K+ ions and the myoendothelial gap junctions (MEGJ). First, an increase of the cytosolic calcium concentration in ECs, either through the spontaneous opening of the transient receptor potential cation channels (TRPV4 and TRPC), allowing the influx of calcium from the extracellular space, or via its release from the sarcoplasmic reticulum (SR) through the inositol trisphosphate receptors (IP3R), gives rise to the activation of the endothelial small-conductance and intermediate-conductance calcium-activated K+ channels, IKCa and SKCa, respectively. Opening of KCa channels brings about the efflux of K+ ions and the activation of the Na+/K+-ATPase as well as the activation of the inward-rectifier K+ (Kir) channels resulting in the decrease of the VSM cell membrane potential. The calcium-sensing receptors (CaSR) neighboring on KCa are responsible for tuning the activity of the KCa channels only within the range of the cytosolic calcium [Ca2+]c, up to ~ 1 mM, the concentration at which their inhibition takes place. The conduction of the hyperpolarization is also enabled by the MEGJ. Apparently, there is an inverse relationship between the vessel size and the number of the MEGJ; i.e., smaller vessels are richer in the MEGJ. The hyperpolarization of the VSM cells inhibits the Cav1.2 (L-type) calcium channels and promotes VSM cell relaxation. *Other abbreviations*: CICR, calcium-induced calcium-release; EEGJ, gap junctions between adjacent endothelial cells; Na+/K+-ATPase, sodium/potassium ATPase

Last but not the least, the IK_Ca_- and SK_Ca_- hyperpolarization was well demonstrated ex vivo. In vivo experiments demonstrated electrical coupling between endothelium and VSM cells, but the tight myoendothelial coupling remains not clearly confirmed [41], and might be only activated under special (pathological?) conditions [208]. Analogous doubts exist about the crucial role of the connexin Cx40 in EDH signaling under in vivo conditions [18, 209]*.* Possible explanation could lie in the different size of the vessels studied in ex vivo (in general, vessels > 100 μm) and in vivo (smaller vessels studied with intravital microscopy) experiments [208]. It is noteworthy that it is the microcirculation that determines the vascular resistance. The EDH is summarized in Fig. 7.

## Vasodilatory stimuli

The endothelium and the VSM cells are exposed to a large variety of vasoactive stimuli, both vasodilatory and vasoconstrictive. The vasoactive stimuli can be divided into physical (subcategorized as mechanical or thermal ) and chemical. For the vascular response, the resulting changes in the membrane potential and the traffic of Ca^2+^ ions are decisive. As mentioned above, the vasodilatation is promoted by the [Ca^2+^]_c_ decrease in the VSM cells and its increase in the endothelial cells, and also by the membrane hyperpolarization in both.

### Mechanical stimuli

The mechanical stimulation is always active even under the resting conditions as a result of the blood flow and physiological blood pressure. The fluid shear stress and pulsatile stretch of the vascular wall continuously stimulate NO production with vasodilatation and prevent the transcription of the atherogenic factors. NO production decreases when the blood flow velocity decreases and when the laminar flow becomes turbulent [35, 235]. The receptors for angiotensin II are involved in the detection of the mechanical stimuli through the activation of the GPCR of the G_q_ type and the PLC/IP_3_ + DAG pathway. There is a vast amount of literature data on AT_1a_ receptors [151], which dominates in the vasculature, but some reports on AT_2_ have also been released [14].

The mechanism of the physiological blood flow-mediated vasodilatation is complex. The blood-flow-induced shear stress induces eNOS expression and subsequent NO release [190, 245]. The stress also induces posttranslational phosphorylation of eNOS [91] and triggers the autocrine production of bradykinin. The latter stimulates further release of NO [80] through the GPCR of the G_q_ type, and this effect may be governed by the activation of the AT_2_ receptors in some vascular beds, as reported for the rat carotid artery [14]. Increased blood flow due to physical activity also results in upregulation of eNOS and vasodilatation. Regular exercise is generally regarded as beneficial for the endothelial function and cardiovascular system [169, 222]. In response to elevated shear stress, the endothelial TRPV_4_ [152], IK_Ca_, and SK_Ca_ [218] channels open, which indicates an increase in endothelial [Ca^2+^]_c_. The epoxygenases of the CYP2C family and the production of prostacyclin are involved as well. Noteworthy, the CYP2C9 is, in addition, linked to the blood flow-induced-vasodilatation which is NO- and prostacyclin-independent [59].

###  Thermal stimuli

In general, temperature rise or its mild decrease induce vasodilatation. Various TRP channels are thought to be temperature-sensitive. The TRPC_5_ channels are highly sensitive to the temperature range of 37–25 °C. Out of the physiological range, the TRPV_1_ channels open near 40 °C and the TRPV_2_ channels are activated at temperatures higher than 50 °C.

Accordingly, a mild hypothermia of dog vessels resulted in vasodilatation with the release of endothelial PGI_2_ and NO and involvement of the muscarinic M_1_ receptors. The latter indicates a local release of acetylcholine. Vasodilatation involving acetylcholine may become more important under hypertension, and possibly also under other pathological conditions [55, 264]. In contrast, a vasoconstrictive response is initiated once the temperature drops below approximately 30 °C. In these cases, the role of endothelium is minor or lacking [55].

### Chemical stimuli

Chemical stimuli can act on endothelium, on VSM or both sites. These stimuli can target both intracellular and plasma membrane structures, principally the GPCR or the ion channels. In the case of GPCR, vasodilatation can be triggered by the activation of the endothelial G_q_, G_s_, and G_i_ receptors and the G_s_ receptors of the smooth muscle. Acetylcholin (muscarinic receptors), bradykinin (B_2_ receptors), histamin (H_1_ receptors), substance P (NK_1_ receptors), and 17β-estradiol (membrane G protein-coupled estrogen receptors GPER) [183, 184, 332, 333] can serve as examples of G_q_ agonists. Examples of endothelial agonists at G_s_ receptors are agonists of β_2_ receptors, PGE_2_ (EP_2_ receptors), and vasopressin (V_2_ receptors). Finally, ET-1 (ET_B_ receptors), agonists of α_2_ receptors, and serotonin (cerebral 5-HT_1_ receptors) can be given as examples of the endothelial G_i_ agonists [113, 235]. In contrast, the activation of the endothelial G_12/13_ and the smooth muscle G_q_, G_i_, and G_12/13_ receptors results in vasoconstriction. As indicated above, the same receptor types can be located on the endothelium, the smooth muscle, or both sites. For example, the muscarinic M_3_ receptors (G_q_) can be found on both the endothelium and the smooth muscle. Acetylcholine induces dose-dependent vasodilatation only when a functional endothelium is present. If not, acetylcholine in low doses has no effect or gives rise to vasoconstriction in high doses. This is widely used as a test of endothelial integrity in ex vivo experiments. The concentration achieved represents another important factor. Thus, acetylcholine can act on endothelial M_3_ receptors at lower concentrations and result in a dilatation, while at higher concentrations, M_1_(?) receptors on VSM are activated resulting in a vasoconstriction.

The effect of a substance may be mediated by multiple receptor types and, in addition, these receptors may be linked to multiple signaling pathways. The complexity of the vasoactive action can be demonstrated on 5-HT. Serotonin has 5-HT_1_, 5-HT_2_ a 5-HT_7_ vascular receptors. At doses > ~ 1 nM (in ex vivo experiments) and under pathological conditions, the substance displays vasoconstrictive effects by the direct action on the muscular 5-HT_2_ (G_q_) receptors. In the CNS, the 5-HT_1_ (G_i_) receptors participate in a similar way (notably, triptan-mediated vasoconstriction in migraine treatment is the result of their activation) [233]. In contrast, at low concentrations and under physiological conditions, 5-HT gives rise to vasodilatation via the endothelial 5-HT_2_ (G_q_) receptors; the 5-HT_7_ (G_s_) receptors may also be involved.

Other vasodilatory substances influence the membrane ion channels directly (e.g., a blocker of Ca_v_1.2 channels, nifedipine) or target the intracellular signaling cascades, such as cyclases (an activator of adenylate cyclase, forskolin, or of soluble guanylate cyclase, riociguat), protein kinases (an inhibitor of PKC, ruboxistaurin, or of ROCK, fasudil), phosphodiesterases (an inhibitor of PDE_3_, cilostazol, or of PDE_5_, sildenafil), ion channels on the SR (an inhibitor of RyR channels, dantrolene), SERCA (an activator, CDN1163), or specific proteins, such as caveolin-1 (binding of cavnoxin results in maintaining the eNOS active).

To date, a number of vasoactive substances are known. Endogenous substances, such as neurotransmitters, hormones, ions, and small gasotransmitters, act as important signaling molecules. The family of exogenous substances ranges from toxins to clinically used drugs. New structures and their targets are constantly being discovered, sometimes unexpectedly, such as vascular bitter taste receptors and their ligands [32, 144].

## Conclusions

Adequate blood supply according to immediate needs is secured by a delicate balance between vasodilatation and vasoconstriction. Imbalance in one (or both) can result in a dysfunction or even a disease. As detailed in this review, the vascular homeostasis is regulated by a plethora of mechanisms, which complement each other and can, if necessary, partially substitute each other. The overall interplay of all participants still remains not fully understood, and deserves further attention.

## Data Availability

No datasets were generated or analysed during the current study.
