# Correction to: The machinery of healthy vasodilatation: an overview

**DOI:** 10.1007/s00424-025-03102-7

**Published:** 2025-06-20

**Authors:** Jana Pourová, Patrícia Dias, Milan Pour, Přemysl Mladěnka

**Affiliations:** 1 Department of Pharmacology and Toxicology, Faculty of Pharmacy, Charles University, Akademika Heyrovskeho 1203, 500 05 Hradec Kralove, Czech Republic; 2 Frick Center for Heart Failure and Arrhythmia, College of Medicine, Dorothy M. Davis Heart and Lung Research Institute, Ohio State University Wexner Medical Center, Columbus, OH USA; 3 Division of Pharmaceutics and Pharmacology, College of Pharmacy, Ohio State University, Columbus, OH USA; 4 Department of Organic and Bioorganic Chemistry, Faculty of Pharmacy, Charles University, Akademika Heyrovskeho 1203, 500 05 Hradec Kralove, Czech Republic

**Correction to: Pflügers Archiv - European Journal of Physiology**

10.1007/s00424-025-03096-2

In the original version of the manuscript, there are 9 incorrect cross references located at page 2, 8, 9, 11, and 12.

The below should be the correct cross-reference of the paper.

- Page 2: (see Sect. 4.6.) should be (see Sect. Endothelium-dependent hyperpolarization (EDH))
- Page 8: (see Sect. 4.2.) should be (see Sect. H2O2)
- Page 9: (see Sect. 3.2.) should be (see Sect. Protein kinase G)
- Page 9: (see Sect. 3.3.) should be (see Sect. Ca2+ sparks and waves in the VSM cells)
- Page 9: (see Sect. 4.2.2.) should be (see Sect. Epoxyeicosatrienoic acids)
- Page 11: (see Sect. 4.6.) should be (see Sect. Endothelium-dependent hyperpolarization)
- Page 11: (see Sect. 3.2.) should be (see Sect. Protein kinase G)
- Page 12: (see Sect. 4.6.) should be (see Sect. Endothelium-dependent hyperpolarization)
- Page 12: (see Sect. 3.1.) should be (see Sect. Protein kinase A)

The original article has been corrected.

